# Application of UHPLC-QqQ-MS/MS Method for Quantification of Beta-Adrenergic Blocking Agents (β-Blockers) in Human Postmortem Specimens

**DOI:** 10.3390/molecules29194585

**Published:** 2024-09-27

**Authors:** Paweł Szpot, Kaja Tusiewicz, Olga Wachełko, Marcin Zawadzki

**Affiliations:** 1 Department of Forensic Medicine, Wroclaw Medical University, 4 J. Mikulicza-Radeckiego Street, 50345 Wroclaw, Poland; 2 Institute of Toxicology Research, 45 Kasztanowa Street, 55093 Borowa, Poland; 3 Faculty of Medicine, Department of Social Sciences and Infectious Diseases, Wroclaw University of Science and Technology, 27 Wybrzeże Wyspiańskiego, 50370 Wroclaw, Poland

**Keywords:** betablockers, beta-adrenergic receptor antagonists, forensic toxicology, drugs intoxication, UHPLC-QqQ-MS/MS

## Abstract

Betablockers are one of the most frequently used medications in cardiology. They can lead to fatal drops in blood pressure and heart rhythm disturbances. Death is functional, and poisoning with this group of drugs can be difficult to detect. The liquid–liquid extraction (LLE) method developed using ethyl acetate at pH 9 successfully identified 18 β-blockers in human blood. The method’s limit of quantification (LOQ) was in the range of 0.1 to 0.5 ng/mL. No carryover of substances between samples was detected, and no interfering ion current signals were observed in the biological samples at the retention times of the compounds or internal standards. All compounds had a coefficient of determination (R^2^) above 0.995. Intraday and interday precision (RSD%) and accuracy (RE%) for low and high QC levels were within 1.7–12.3% and −14.4 to 14.1%, respectively. Very good recovery (80.0–119.6%) and matrix effect (±20.0%) values were achieved for all compounds. In addition, fragmentation spectra were collected for all the examined substances, and high-resolution spectra were presented for landiolol and metipranolol, because they are not available in commercial HRMS spectra databases. The developed method was applied in authentic postmortem samples.

## 1. Introduction

Betablockers (β-blockers), as antagonists to β-adrenergic receptors, are pharmacological drugs crucial in managing heart rate, blood pressure, and airway reactivity [1]. The compounds representing this group of cardiovascular drugs are highly heterogeneous, demonstrating varying levels of potency, selectivity, and duration of action or tissue solubility [2]. The classification of adrenergic receptors into alpha (α) and beta (β) types, along with the identification of β-adrenergic receptors subtypes (β1 mainly in the heart and β2 responsible for vascular and airway relaxation), led to the differentiation of three generations of betablockers. First-generation β-blockers, such as propranolol, are non-selective, affecting both β1 and β2 receptors. Second-generation β-blockers, such as practolol, focus on cardioselectivity (they have higher selectivity towards β1 receptors than β2), which is dose-dependent. Third-generation β-blockers, including carvedilol, labetalol, and nebivolol, have additional properties, such as α1-adrenergic receptor antagonism and nitric oxide release [1,3]. All β-blockers, due to their structural similarity to catecholamines such as adrenaline and noradrenaline, competitively inhibit adrenergic stimulation by binding to β-adrenergic receptors and blocking agonist activity, thereby reducing sympathetic nervous system activity [1,2,3].

These pharmacological actions underscore their effectiveness in managing, beyond others, hypertension, heart failure, and ischemic heart disease. Especially, an umbrella review performed by Ziff et al. [4] indicates a particularly beneficial effect of β-blockers in cases of heart failure and in people with reduced ejection fraction with sinus rhythm. Regarding the treatment of hypertension, review articles and meta-analyses [5,6,7] highlight the need for caution, particularly in cases of primary hypertension and among elderly individuals. In these instances, it is advised not to use β-blockers as a first-line therapy and to consider the heterogeneity within this class of medications. However, some studies suggest the potential use of β-blockers in the treatment of hypertension in particular cases, including in elderly patients [8,9].

The effects of β-blockers on foetal outcomes can be controversial. These substances cross the placenta and may cause physiologic changes in the foetus. However, it should be taken into account that the betablockers, when used individually during pregnancy, can bring significant improvement in pregnant women with hypertension [10,11].

β-blockers can be particularly dangerous in cases of asthma, atrioventricular block, or betablocker intolerance. Furthermore, there are a number of side effects associated with the use of these cardiological drugs, including headache, dizziness, fatigue, heart block, bradycardia, and hypoglycaemia [9].

The remarkably widespread use of β-blockers in medicine makes it necessary to monitor the presence and concentration of these drugs in biological material for forensic toxicology. Not only because of, for example, postmortem confirmation of the ingestion of a particular group of drugs and their possible contribution to functional death, but also because of reported suicidal intentional poisonings with betablockers. Ayhan et al. [12] reported that in suicidal cardiac drug poisonings in the emergency departments they, up to 37% of cases were related to β-blockers use. The literature describes cases of β-blocker poisonings [13,14], some of which were classified as suicide poisonings, with both non-fatal [15,16,17,18] and fatal [19] outcomes.

Of particular note, from the point of view of forensic toxicology, is the fact that cardiac drug poisonings tend to be mixed poisonings. A prospective cohort analysis of 280 cases of β-blockers exposure by Love et al. [20] shows that approximately 31% of poisonings involved mixed cardiovascular drug poisonings. This together with the fact that the therapeutic concentration of β-blockers is very low (0.001–5 µg/mL, depending on the compound [21]) requires the availability of sensitive and specific analytical methods in the toxicology laboratory that can be used to analyse such a complex matrix as biological material, especially postmortem samples. In the case of toxic (0.12–16 µg/mL) and lethal (0.17–142 µg/mL) concentrations, attention should be paid to the fact of the short biological half-time for most compounds in the β-blockers group [21], which also implies that low concentrations of these compounds may need to be determined to confirm betablocker uptake. It is especially crucial in clinical cases where a person is in ward and antemortem samples are not tested, there occurs elimination of xenobiotics over time with urine, and then, postmortem samples require extremely sensitive methods to detect compounds that could contribute to death [22].

Among the methods developed to date for the determination of β-blockers, it is worth mentioning those utilizing capillary electrochromatography coupled with mass spectrometry (MS) [23,24], matrix-assisted laser desorption (MALDI) [25], high-performance liquid chromatography with ultraviolet detection (HPLC-UV) [26,27] and HPLC with fluorometric detection [28], as well as gas chromatography coupled with mass spectrometry (GC-MS) [29,30,31]. Among liquid chromatographic methods, various analytical approaches were described, including the utilization of reversed-phase ion-pair HPLC-UV detection [32], the dispersive liquid–liquid extraction (LLE) procedure [33], automated in-tube solid-phase microextraction (SPME) [34], and derivatization with dabsyl chloride [35].

In the case of examinations of very complex biological matrices, the most preferable analytical method to date is liquid chromatography coupled with mass spectrometry [36,37], which provides both high sensitivity and selectivity of analyses. In light of the growing detection of betablockers, the introduction of new compounds in this class, and an increasing number of cases involving their effects on death (such as medical errors related to their use or inadequate care in nursing homes, as well as potential links to miscarriage or suicidal poisonings), the authors decided to develop a method for determining these substances in postmortem material and to emphasize their toxicological and forensic implications.

The aim of the study was to develop a sensitive and selective method for the determination of compounds from the β-blockers group in biological material. Furthermore, the method was applied to authentic cases, which included this cardiovascular drug intake. Finally, the forensic aspect of betablocker intoxication expertise was extensively discussed. Another goal was to highlight an important aspect, in the authors’ view, regarding the inclusion of beta-adrenergic blocking agents (one of the most commonly prescribed drug groups) in routine toxicological examinations. Despite being considered safe, these agents can significantly impact investigations. To date, to the authors’ knowledge, no articles have been published that address the forensic toxicology aspects of this class of compounds, particularly in relation to their diverse chemical, pharmacological, and pharmacokinetic properties.

## 2. Results

**Figure 1:**
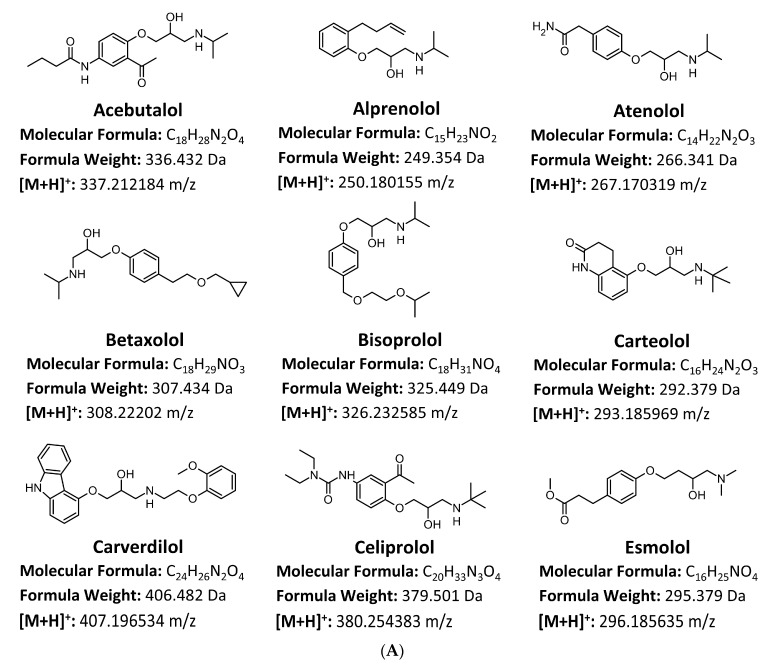

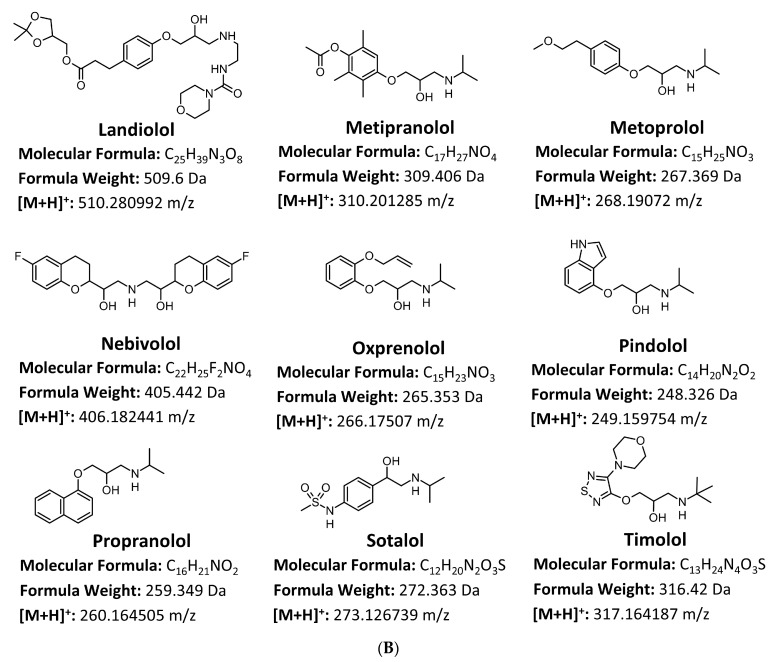
(**A**). Structures of determined compounds. (**B**). Structures of determined compounds.

The simple, selective, and very sensitive method for the detection of 18 β-blockers (structures are shown in Figure 1A,B) was developed and fully validated in accordance with Scientific Working Group for Forensic Toxicology (SWGTOX) recommendations. Furthermore, analysis was performed using the lowest sample volume to date (100 µL) for various biological matrix (blood, putrefaction fluid, gastric contents, and solid tissue homogenates), which is a significant advantage, especially in forensic toxicology examinations, due to the need to perform many different tests on the unique sample collected in each case. The quantitation of 18 β-blockers was performed using the internal standard addition method with 3 deuterated β-blocker analogues (atenolol-d_7_, metoprolol-d_7_, and propranolol-d_7_). The determination of the investigated substances was carried out in the multiple reaction monitoring (MRM) mode. A summary of precursor and product ions, collision energies, dwell time, Q1-Q3 pre-bias voltages, and retention time for each compound is presented in Table 1.

**Figure 2:**
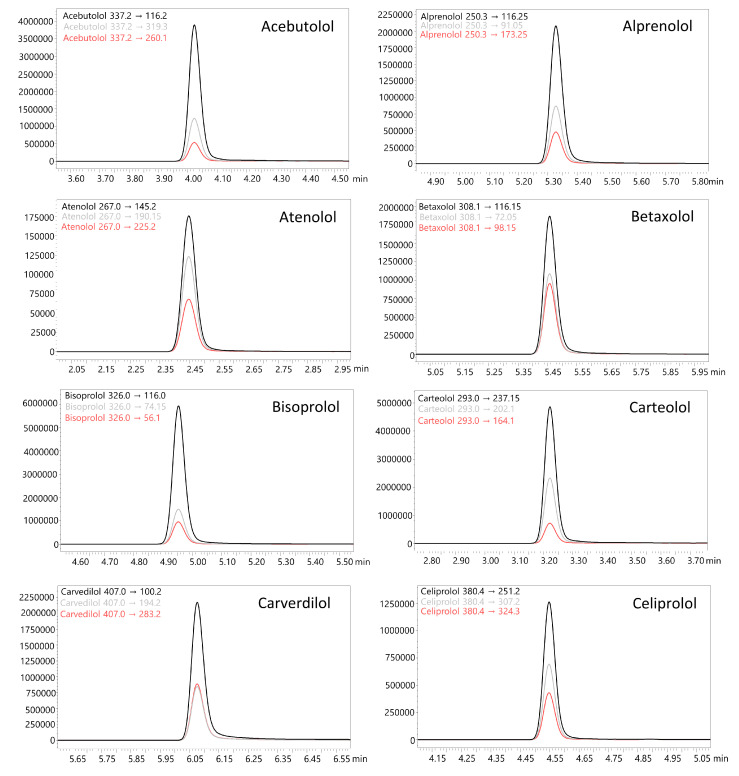
Multiple reaction monitoring (MRM) chromatograms of determined β-blockers at a concentration of 50 ng/mL (whole blood matrix).

**Figure 3:**
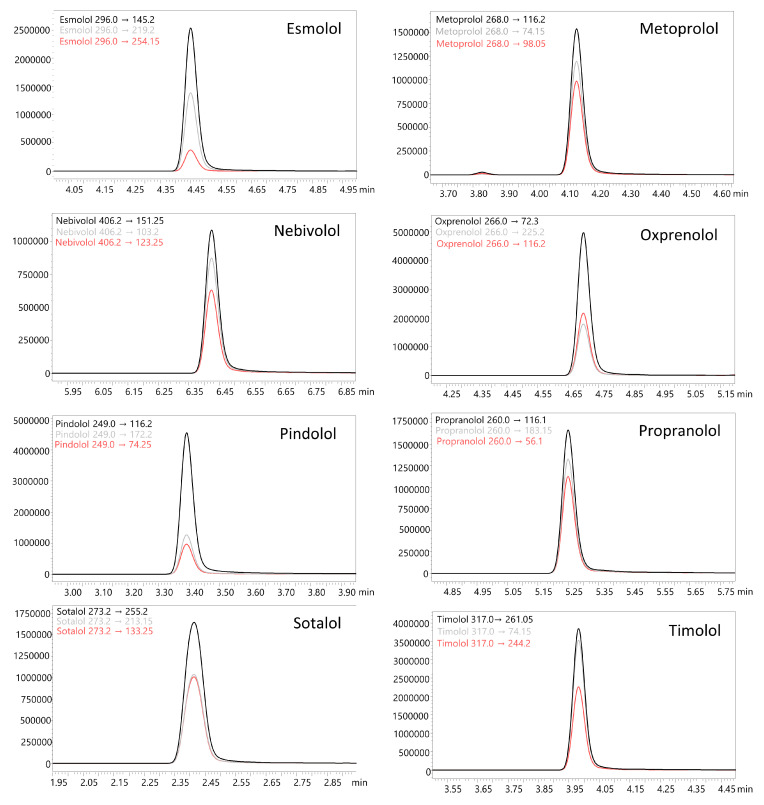
Multiple reaction monitoring (MRM) chromatograms of determined β-blockers at a concentration of 50 ng/mL (whole blood matrix).

**Figure 4:**
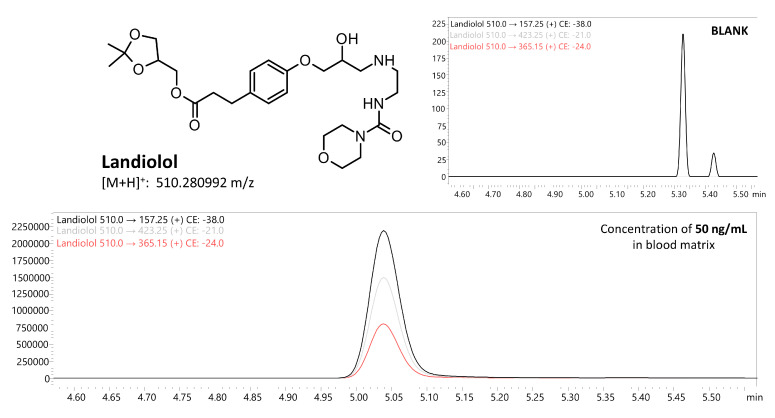
Multiple reaction monitoring (MRM) chromatograms of blank and landiolol at a concentration of 50 ng/mL (whole blood matrix).

**Figure 5:**
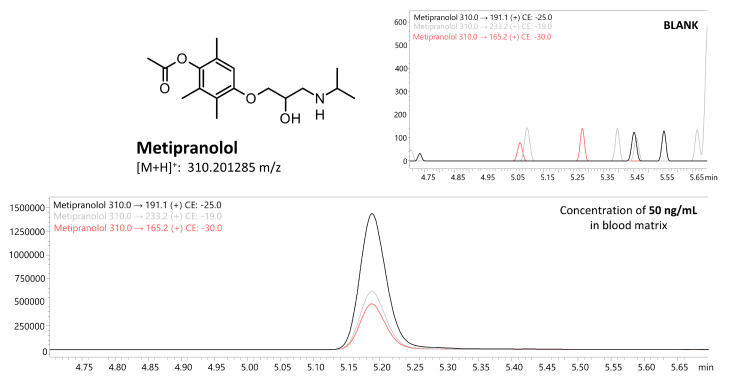
Multiple reaction monitoring (MRM) chromatograms of blank and metipranolol at a concentration of 50 ng/mL (whole blood matrix).

**Figure 6:**
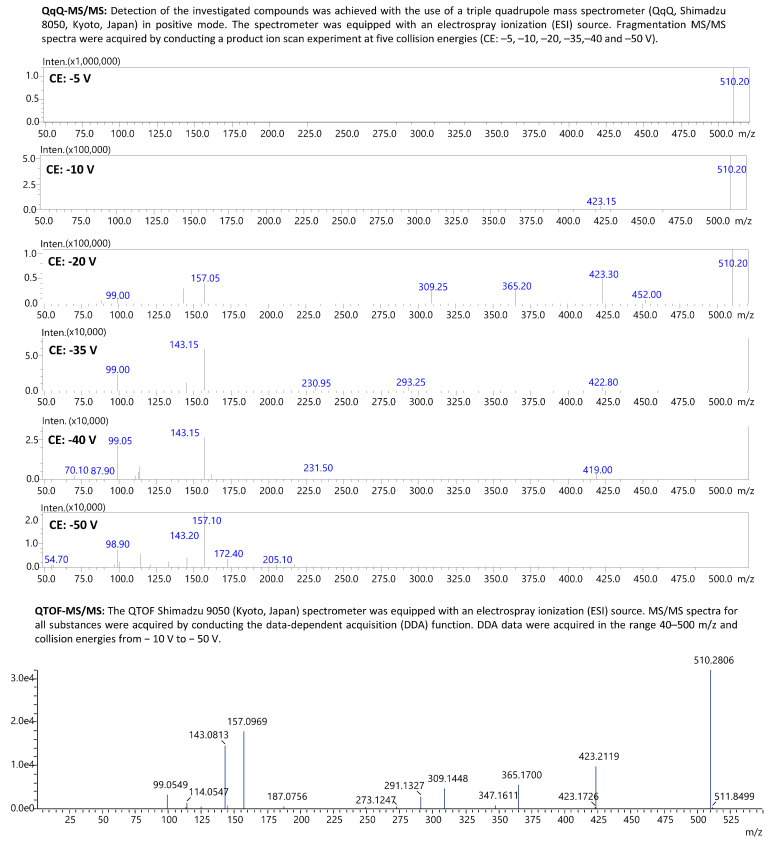
Fragmentation spectra (MS/MS) of landiolol achieved with the use of QqQ-MS and QTOF-MS.

**Figure 7:**
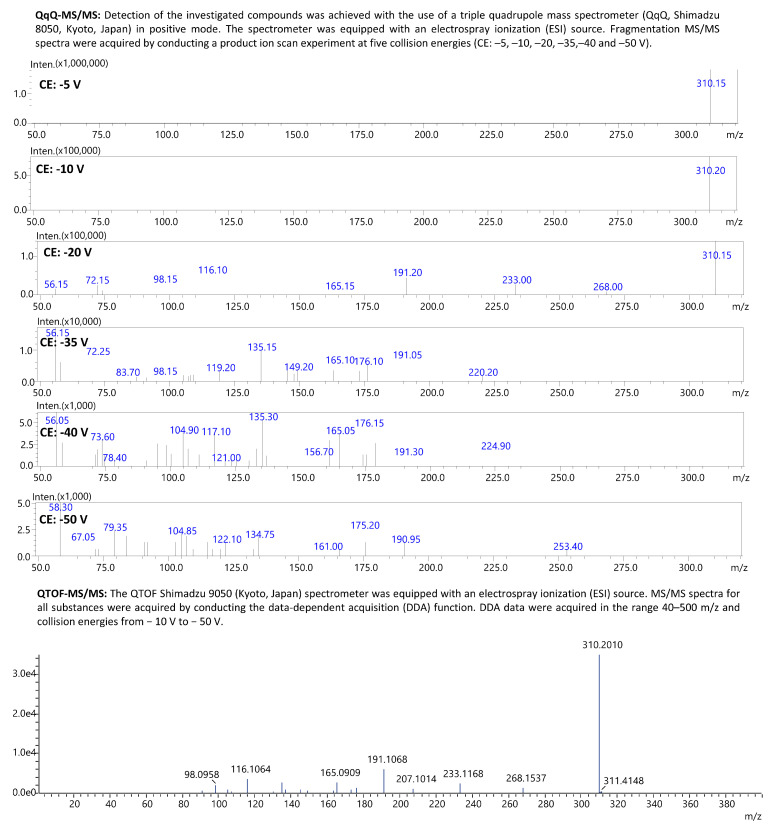
Fragmentation spectra (MS/MS) of metipranolol achieved with the use of QqQ-MS and QTOF-MS.

The liquid–liquid extraction (LLE) method developed using ethyl acetate at pH 9 successfully identified 18 β-blockers in biological fluids (blood, putrefaction fluid, and gastric contents) and solid tissues (foetal soft tissues, kidney, and liver). To establish the calibration curve range and determine the correlation coefficient, 12 calibration points were prepared in whole blood and analysed using the developed UHPLC-QqQ-MS/MS method. Values that exceeded the acceptable criteria (e.g., saturated levels) were subsequently excluded, ensuring that a minimum of 6 valid calibration points were included in each analysis. The method’s limit of quantification (LOQ) for acebutolol, bisoprolol, pindolol, and timolol was 0.1 ng/mL; oxprenolol, propranolol, alprenolol, carvedilol, celiprolol, esmolol, metoprolol, and nebivolol was 0.2 ng/mL; and landiolol, metipranolol, atenolol, betaxolol, carteolol, and sotalol was 0.5 ng/mL. No carryover of substances between samples was detected, and no interfering ion current signals were observed in the biological samples at the retention times of the compounds or internal standards. In forensic toxicology, samples may exceed the method’s upper quantification limit, particularly in fatal cases [38]. Here, the dilution effect parameter offers insight into the method’s suitability. In the case of our method, the values did not exceed ±10% for 10-fold dilution. All compounds had a coefficient of determination (R²) above 0.995. Intraday and interday precision (RSD%) and accuracy (RE%) for low and high QC levels were within 1.7–12.3% and ±14.4%, respectively. Very good recovery (80.0–119.6%) and matrix effect (±20.0%) values were achieved for all compounds. A summary of validation results is available in Table S1 (Supplementary Materials). Multiple reaction monitoring (MRM) chromatograms for the substances at a concentration of 50 ng/mL are presented in Figure 2 and Figure 3. MRM chromatograms for blank samples and those at the LOQ level for each substance are provided in Figure S1 (Supplementary Materials). In addition, fragmentation spectra (presented in the Supplementary Materials) were collected for all the examined substances. For landiolol and metipranolol, high-resolution spectra are not available in commercial HRMS spectra databases (e.g., mzCloud™). We conducted QTOF analysis to complement the existing data, thereby providing comprehensive fragmentation spectra for these two compounds, which will be valuable for future reference in analytical studies. In the case of abovementioned substances, MRM chromatograms of a blank sample, a sample at a concentration of 50 ng/mL, as well as QqQ product ion scan spectra and QTOF mass spectra are shown in Figure 4, Figure 5, Figure 6 and Figure 7. Product ion scan experiments conducted using a triple quadrupole at six collision energies (−5, −10, −20, −35, −40, and −50 V) have been also presented in the aforementioned figures (as well as in the Supplementary Materials). These experiments may assist researchers in the preliminary identification of these compounds in biological samples, even in the absence of reference standards.

### Toxicological Analysis of Authentic Biological Samples

The developed method was utilized for the quantitative determination of selected betablockers in samples secured during autopsies, as requested by the prosecutor. We have decided to briefly describe a few cases in which betablockers were detected.

The first case (Case 1) involves the remains of a foetus (IV/V month of gestation) found at a wastewater treatment plant. “Foetal soft tissues” were secured for testing, and prior to toxicological analysis, these tissues were homogenised using ultrasound. The second case (Case 2) pertains to a young woman found deceased in her apartment as a result of asphyxiation. Putrefaction fluid, kidney, and liver samples were examined. The third case (Case 3) concerns a man who died by suicide through drug poisoning. In this case, blood and gastric contents were secured for analysis. The fourth case (Case 4) involves a woman who was suspected of drug poisoning. Blood was sent to our laboratory for testing. In this case, no additional details are known.

**Figure 8:**
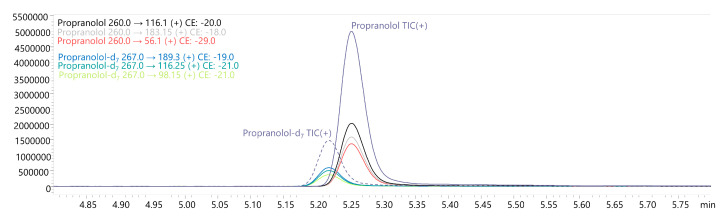
Chromatogram of authentic case sample (Case 2; kidney).

In Case 1, toxicological analysis of foetal soft tissues revealed bisoprolol at a concentration of 1 ng/g and metoprolol at 5.3 ng/g. Case 2 exhibited the following concentrations of propranolol in the secured samples: 99.0 ng/mL (putrefaction fluid), 555 ng/g (kidney), and 2610 ng/g (liver). In Case 3, metoprolol was determined at a concentration of 103 ng/mL and >10,000 ng/mL in the blood and gastric contents, respectively. What is more, propafenone was present at a concentration of 3187 ng/mL (blood) and >10,000 ng/mL (gastric contents). Finally, in Case 4, the blood test results exhibited metoprolol at a concentration of >10,000 ng/mL. The chromatogram of the authentic kidney sample from Case 2 depicting the spectrum of propranolol and the internal standard is shown in Figure 8.

## 3. Discussion

### 3.1. Overview of the Determination of β-Blockers in Biological Samples

The summary of liquid chromatography methods coupled with mass spectrometry applied for the determination of β-blockers in biological samples is presented in Table 2. In most methods, the biological samples examined were serum, urine, or plasma [39,40,41,42,43,44,45], and sample volumes were in the range of 200 µL [45] to 5 mL [40]. In toxicological analysis of human postmortem samples, obtaining a large volume of biological material is often challenging. This presents two primary issues: first, there is often only a small quantity of blood available for testing, particularly in cases of significant blood loss (such as accidents) or when examining neonate samples [46]. Second, multiple tests are usually required from a single vial of blood, necessitating methods that use minimal amounts of material for each test. Furthermore, in forensic toxicology, plasma or serum is rarely available due to the decomposition of biological materials and blood haemolysis. Consequently, it is essential to have a precise, sensitive, and accurate method for detecting xenobiotics in postmortem biological fluids and tissues during toxicological examinations [47]. Out of all summarised methods, only Dupuis et al. [48] described a method specifically designed for forensic applications; however, the sample volume was 20-fold larger than in our method.

When developing a method, it is crucial to avoid using compounds that are present as pharmaceuticals in medical treatments (such as sulfapyridine, pindolol etc.) [41,45] as internal standards, as this can lead to inaccurate quantitative analysis results. Consequently, substances that may already be present in postmortem or antemortem biological material should not be used as internal standards. The recommended approach is to use deuterated derivatives of the analysed substances. Among all the studies reviewed, deuterated analogues were utilised in three papers, including one with metoprolol-d_7_ [39]. However, our paper is the first to develop a protocol that incorporates deuterated internal standards with varying chemical structures (from β-blockers group), enhancing the method’s precision and reliability.

Solid-phase extraction (SPE), which has been applied in three studies [42,43,44], can be considered as a very good choice for sample purification, because it selectively binds substances intended for further analysis, resulting in very clean extracts. However, SPE has drawbacks, including a lengthy sample preparation time, the need for a vacuum-equipped SPE extraction cabinet, and the consumability of the columns. While this approach is advantageous for experimental studies, it becomes problematic when dealing with large number of samples, as commonly seen in toxicology and clinical laboratories, due to increased costs and longer analysis times. Therefore, many researchers have chosen liquid–liquid extraction (LLE) with different organic solvents, an optimised procedure that has been successfully applied for analysis.

Among the developed methods, the limits of quantification (LOQ) ranged from 0.1 ng/mL [42] to 10.0 ng/mL [41,48]. These results were primarily achieved through complex sample preparation procedures (including derivatization) and the use of large volumes of biological fluids and injection volumes. Although large injection volumes are a straightforward method to enhance analytical sensitivity, they can lead to clogged chromatographic columns with small particle sizes, reducing column lifetime. Additionally, large injection volumes introduce more matrix into the detector, causing ionisation competition between matrix ions and analyte ions, which increases background noise and hinders the achievement of low LOQ values. In the case of urine samples, hydrolysis with β-glucuronidase was also performed for achieving lower limits of quantification [40].

### 3.2. Forensic Aspects

Poisoning by beta-adrenergic receptor antagonists (betablockers) can lead to significant, and sometimes fatal, drops in blood pressure and disturbances in heart rhythm. Death in these cases is functional in nature, and poisonings by this group of drugs can be challenging to detect in a hospital setting. This difficulty arises because routine diagnostic tests in hospitals often focus primarily on detecting drugs and substances with high toxicity, such as opioids, benzodiazepines, amphetamines, barbiturates, and tricyclic antidepressants. Standard toxicology screening panels do not detect cardiological drugs. Fortunately, due to the functional nature of these poisonings, standard procedures, including symptomatic treatment such as gastric lavage, the use of endocardial electrodes, and haemodiafiltration, often prove effective, and survival has been reported even in individuals with blood concentrations reaching several tens of µg/mL [49].

The postmortem diagnosis of betablocker poisoning is highly complex and case-dependent. The first type of these cases involves biological material from patients who were admitted to the hospital but did not survive, despite resuscitation efforts. In such situations, forensic toxicologists usually have access to the patient’s medical records and can suspect betablocker poisoning based on the symptoms, allowing for targeted testing. This scenario is markedly different from cases where only biological material from a deceased individual is sent to the toxicology laboratory, particularly if the person was found at home, in a park, etc., without circumstances suggesting betablocker poisoning. In most toxicology laboratories, only highly toxic drugs and substances are typically detected. Even if the laboratory has appropriate methods for detecting betablocker drugs, due to the functional nature of the poisoning, it can be challenging to conclusively prove that these substances contributed to death. Exceptions may include cases where clear evidence is found, such as suicide notes, witness’ statements, a large number of tablets in the gastrointestinal tract, or extremely high blood concentrations of betablockers.

From a forensic toxicology perspective, several key aspects should be noted: betablockers are generally well-tolerated and safe medications, but symptoms of poisoning can sometimes appear even at concentrations slightly above therapeutic levels. Patients with hypertension do not always adhere to medical guidelines and may take these medications on their own, which is why some of them (e.g., Sotalol) are monitored through therapeutic drug monitoring (TDM). Substances from the betablocker group are not frequently detected in forensic toxicological analyses, despite being among the most commonly prescribed medications. This may be due to several reasons, as follows:

1. In some laboratories, immunoenzymatic screening tests (ELISA) are performed for a few commonly abused drug groups (betablockers are not among them), followed by confirmatory analyses targeted at substances from groups that yielded positive results in the screening using LC-MS or GC-MS. The use of ELISA-based screening methods is associated with insufficient specificity and sensitivity, leading to fewer positive results than would be expected. Moreover, even the application of GC-MS may not result in the detection of all betablockers at ng/mL concentrations [50].
2. The sensitivity of the analytical methods used is another important aspect regarding the frequency of betablocker detection in forensic toxicological studies. Betablockers with an average therapeutic concentration below 100 ng/mL were detected less frequently (33% positive results) than betablockers with an average therapeutic concentration above 500 ng/mL (70% positive results) [51]. The developed method demonstrates high sensitivity, with limits of quantification (LOQ) ranging from 0.1 to 0.5 ng/mL. This significantly enhances the likelihood of detecting betablockers, even in trace amounts, in blood samples.
3. The selected pharmacokinetic parameters vary significantly: half-life (e.g., structurally similar ultra-fast-acting β1-adrenoreceptor antagonists landiolol and esmolol have a T_1/2_ of 2–8 min and 3–16 min, respectively [49]), lipophilicity, varying degrees of protein binding (e.g., acebutolol [10%]—propranolol [95%]), and different volumes of distribution (e.g., atenolol [0.7 L/kg]—nebivolol [10 L/kg]). These parameters affect the postmortem redistribution of betablockers and complicate the interpretation of forensic toxicological results [52]. For example, labetalol, propranolol, and carvedilol have a large volume of distribution, are largely protein-bound, undergo significant hepatic metabolism, have negligible renal clearance, and do not require dose adjustment in chronic kidney disease, while sotalol, nadolol, and atenolol have entirely opposite characteristics. Their different pharmacological properties influence their clinical effects, such as selectivity for β-1-adrenergic receptors (e.g., metoprolol > propranolol), α-adrenergic receptor antagonist activity (e.g., carvedilol, labetalol), intrinsic sympathomimetic activity (e.g., acebutolol, pindolol), membrane-stabilizing activity (MSA) due to sodium channel blockade (e.g., propranolol, acebutolol, and labetalol), central nervous system depression (e.g., propranolol), and Class III antiarrhythmic effect due to potassium channel antagonism (e.g., sotalol) [53]. Despite the different physicochemical properties, the developed method allows for the simultaneous determination of 18 betablockers in a single analytical run using a single preparation of biological material.
4. Some betablockers are unstable. Few studies have been published on the stability of betablockers in postmortem material. One particularly interesting example in this context is landiolol, which has been found to be highly unstable in biological material. It is difficult to quantify, because it is rapidly hydrolysed by pseudocholinesterase during sample storage. Researchers have attempted to stabilise it by adding enzyme inhibitors, such as pyridostigmine or neostigmine bromide, to the biological material [54]. It is also worth noting that even the mere fact of drawing blood into different tubes containing commercial plasma separators can result in a decrease in the concentration of certain betablockers by several percent [55].
5. The type of biological material tested: For the interpretation of results in a routine forensic toxicological analysis, at least two biological materials are typically used, most often blood and urine/vitreous humour. Blood is collected, because it is the international standard in the diagnosis of sudden death. The determination of xenobiotics in the blood serves, among other things, to interpret the impact of the detected substance on psychomotor functions and allows for the assessment of whether the concentration is therapeutic, toxic, etc. In contrast, the examination of the second biological material (e.g., urine, vitreous humour) provides additional data, such as information on the phase of metabolism and sometimes evidence of chronic use, among others. In forensic toxicology practice, situations arise where these materials are not available: the bladder may have been empty, blood may be unavailable due to complete exsanguination (e.g., in car accidents, plane crashes, deaths under train wheels, or in cases of advanced decomposition). In such cases, additional biological materials such as liver, kidney, or sometimes, only fragments of a muscle (e.g., death in a fire) are used. In a muscle sample from a deceased individual in a plane crash described by Johnson and Lewis 2006 [56], the concentration of propranolol in the muscle was more than ten times lower than in postmortem blood. The developed method can be effectively applied to analyse various types of biological materials, including foetal soft tissues, putrefaction fluid, kidney, liver, and gastric contents. Valuable data can be provided by studies examining the distribution of betablockers. Due to the diversity of this group of compounds, routes of administration should also be considered, for example, timolol administered conjunctivally for glaucoma treatment, as it reduces intraocular pressure. Its blood concentration may, therefore, be very low: 0.13–1.72 ng/mL (our method allows for determining such concentrations) in plasma [57] and undetectable by some methods. The method presented in this paper allows for the determination of timolol in the range of concentrations mentioned, in contrast to the methods presented in Table 2.

Drug interactions are a separate issue entirely. Patients with cardiovascular diseases are often treated with polypharmacy. Due to the functional nature of deaths, it is challenging to find reliable information about fatal poisonings with these substances outside of suicide attempts. This raises the question of whether, despite the therapeutic concentration of the detected compound, the blood pressure might have dropped enough to lead to cardiovascular failure and, consequently, death. Additional confirmation of this hypothesis may come from detecting other antihypertensive drugs, diuretics, etc., in the biological material. However, it is important to remember that detecting vasodilators, such as nitroglycerin, remains challenging, as they are often not routinely measured, and in many cases, are impossible to detect. The combination of calcium channel blockers (e.g., nifedipine) with betablockers can exacerbate hypotension. Taking digitalis glycosides together with β-blockers can prolong atrioventricular conduction. Carvedilol may affect glucose tolerance and mask symptoms associated with hypoglycaemia (e.g., tremors, palpitations, sweating) [58]. For these reasons, particular attention should be paid to the scope of toxicological tests performed on individuals being treated for multiple conditions and on elderly patients.

Typically, the toxicity of betablockers is directly related to the dose; however, it should be noted that certain conditions, such as liver cirrhosis, especially in the case of betablockers that strongly bind to proteins (e.g., carvedilol), can cause toxic effects even when a therapeutic dose is used [59].

## 4. Materials and Methods

### 4.1. Biological Material and Chemicals

Drug-free blank whole blood samples used for the development and validation of the method were selected from our laboratory. The approval of the bioethics committee required has been obtained (consent no. KB-184/2023N). Blank blood samples were screened prior to spiking to ensure that they were free from drugs. Authentic human biological samples were sent to our laboratory for toxicological examination.

The following reagents were used in the development of the method: water (Li-Chrosolv^®^ LC-MS), acetonitrile (LiChrosolv^®^ LC-MS), methanol (LiChrosolv^®^ LC-MS), ethyl acetate (LiChrosolv^®^ LC-MS) were purchased from Sigma-Aldrich (Steinheim, Germany). Acebutolol hydrochloride (10 mg neat) and carvedilol (as free base, 10 mg neat) were purchased from LGC Standards (Teddington, UK). Alprenolol hydrochloride (10 mg neat), betaxolol hydrochloride (10 mg neat), bisoprolol hemifumarate (10 mg neat), nebivolol hydrochloride (10 mg neat), propranolol hydrochloride (10 mg neat), sotalol hydrochloride (10 mg neat), metoprolol (as free base, 1 mg/mL in methanol), oxprenolol hydrochloride (10 mg neat), timolol maleate (1 mg/mL in methanol), atenolol-d_7_ (as a free base, 100 μg/mL in methanol), and propranolol-d_7_ (as free base, 100 μg/mL in methanol) were purchased from Chiron (Trondheim, Norway). Atenolol (as free base 1 mg/mL in acetonitrile), celiprolol hydrochloride (5 mg neat), landiolol hydrochloride (5 mg neat), and metoprolol-d_7_ tartrate (100 μg/mL in methanol) were purchased from Sigma-Aldrich (Steinheim, Germany). Carteolol hydrochloride (5 mg neat), metipranolol hydrochloride (5 mg neat), pindolol (10 mg neat), and esmolol hydrochloride (10 mg neat) were purchased from TRC (Toronto, ON, Canada).

### 4.2. Instrumentation

Analysis was performed using an ultra-high-performance liquid chromatograph (UHPLC, Shimadzu Nexera LC-40; Kyoto, Japan). The chromatographic separation was carried out with the use of a Kinetex XB-C18 (150 × 2.1 mm i.d., particle size 2.6 μm; Phenomenex, Torrance, CA, USA) with the thermostat set at 40 °C. The mobile phase consisted of 0.1% formic acid and 10 mM ammonium formate in water (A) and 0.1% formic acid in acetonitrile (B). The gradient elution was carried out at a constant flow of 0.4 mL/min. The gradient applied was as follows: 0 min, 5% B; 12 min, 98% B; 14 min 98% B; and 15 min, 5% B. Return to the initial gradient compositions (95% A/5% B) was performed for 5 min. The injection volume was 2.0 μL. Detection of the investigated compounds was achieved with the use of a triple quadrupole mass spectrometer (QqQ, Shimadzu 8050, Kyoto, Japan) in positive mode. The spectrometer was equipped with an electrospray ionisation (ESI) source. Determination of the investigated substances was carried out in the multiple reaction monitoring (MRM) mode. The following MS parameters were fixed: nebulising gas flow, 3 L/min; heating gas flow, 10 L/min; interface temperature, 250 °C; desolvation line temperature, 200 °C; heat block temperature, 350 °C; and drying gas flow, 10 L/min. Fragmentation MS/MS spectra were acquired by conducting a product ion scan experiment at five collision energies (CE: –5, –10, –20, –35,–40, and –50 V).

Quadrupole time-of-flight (QTOF) Shimadzu 9050 (Kyoto, Japan) spectrometer was equipped with an electrospray ionisation (ESI) source. The following QTOF parameters were fixed: nebulizing gas flow, 3 L/min; heating gas flow, 10 L/min; interface temperature, 250 °C; desolvation line temperature, 200 °C; interface voltage (+), 4.50 kV; heat block temperature, 350 °C; and drying gas flow, 10 L/min. Analyses were performed using an UHPLC, Shimadzu Nexera LC-40 (Kyoto, Japan) with the same column and gradient conditions as described above. MS/MS spectra were acquired by conducting the data-dependent acquisition (DDA) function. DDA data were acquired in the range 40–500 *m*/*z* and collision energies from −10 V to −50 V.

### 4.3. Sample Preparation

Biological fluids: A volume of 100 μL of biological fluid (blood, putrefaction fluid, and gastric contents) was transferred into 2 mL Eppendorf tubes. Next, 10 μL of methanolic internal standard solution (500 ng/mL) and 200 μL of buffer (pH 9) were added. Liquid–liquid extraction with 1 mL of ethyl acetate was carried out for 5 min. The sample was centrifuged for 5 min (14,000× *g* at 4 °C). The organic phase was then transferred into 2 mL Eppendorf tubes and evaporated to dryness under a stream of inert nitrogen gas at 40 °C. The dry residues were dissolved in 50 μL of methanol. The solution was then transferred into glass inserts of autosampler vials and analysed by UHPLC-QqQ-MS/MS.

Solid tissues: Tissue samples (of foetal soft tissues, kidney, and liver) were prepared for analysis as in [60]. Homogenisation was performed by weighing 1 g of solid sample, which was transferred to a plastic tube (12 mL) and mixed with 1 mL of water (LC-MS grade). The tube was then placed in a glass beaker containing ice cubes, and the contents of the tube were homogenised using a Q55 sonicator (QSonica, Newtown, PA, USA).

### 4.4. Working Solutions, Calibration Curve, and Quality Control Samples

The stock solutions, powders, and standard solutions were stored at –20 °C. Standard solutions of atenolol-d_7_, propranolol-d_7_, and metoprolol-d_7_ serving as an internal standard (IS) were mixed and diluted with methanol to yield a final concentration of 500 ng/mL of each compound. All standards in powder form were dissolved in methanol except for carteolol, which was dissolved in ethanol. From standard solutions of 18 determined compounds, 3 separate mixes (MIX1, MIX2, and MIX3 containing 6 substances each) were initially prepared by diluting with methanol yielding at a final concentration of 100 μg/mL of each compound. Then, the 3 mentioned mixes were combined into 1 by diluting with methanol to a concentration of 10 μg/mL. Such prepared mixes were then further diluted with methanol to obtain working standard solutions at the following concentrations: 1, 2, 5, 10, 50, 100, 200, 500, 1000, 2000, 5000 ng/mL. Calibration points and quality control (QC) samples were prepared by mixing the appropriate working solution with blank whole blood. The final concentrations of the calibrators were 0.1, 0.2, 0.5, 1, 5, 10, 20, 50, 100, 200, 500, 1000 ng/mL for each determined compound in biological material. Quality control samples were prepared by spiking blank human whole blood to yield a final concentration of 5 (low QC) or 200 and 500 (high QC) ng/mL. For quadrupole time-of-flight (QTOF) analysis, analytical standards of the tested compounds (landiolol and metipranolol) were used at a concentration of 100 ng/mL.

### 4.5. Validation Process

Evaluated parameters of the method included examination of selectivity, linearity, precision and accuracy, carryover, limit of quantification, dilution effect, recovery, and matrix effect. Validation was performed similarly to previously published papers [61,62], and all parameters were assessed in accordance with Scientific Working Group for Forensic Toxicology (SWGTOX) recommendations [63]. Evaluated parameters of the method included examination of selectivity, interferences from stable-isotope internal standard, linearity, precision and accuracy, carryover, limit of quantification, recovery, and matrix effect as well as dilution effect. Selectivity: 10 different lots of blank whole blood samples from different origins were tested for possible endogenous interference substances at the retention times of analytes and IS. Interferences from stable-isotope internal standard. The isotopically labelled compounds may contain the non-labelled compound as an impurity. Interferences from the isotopically labelled internal standard were evaluated through the analysis of a blank whole blood sample fortified with IS and monitoring the signal of non-labelled compounds. An unacceptable level of interference was defined to be above the limit of quantification (LOQ) of the method. Precision and accuracy were estimated by replicating analysis (*n* = 5) of QC samples at 2 concentration levels: 5 and 200 ng/mL or 5 and 500 ng/mL (depending on linearity range for each substance). Precision was defined as relative standard deviation (RSD%), while accuracy was expressed as mean relative error (RE%). To investigate the carryover, 3 blank samples without analytes were analysed after the highest calibration sample at the betablocker concentration of 1000 ng/mL. The blood samples containing an internal standard without determined analytes were evaluated for the presence of betablockers. The lower limit of quantification (LLOQ) was defined as the concentration at which the relative standard deviation (RSD%) and relative error (RE%) did not exceed 20% and 15%, respectively. The recovery and matrix effect values were evaluated at each of the 2 concentration levels: 5 (low QC) and 200 (high QC) ng/mL or 5 (low QC) and 500 (high QC) ng/mL. The recovery (in percent, *n* = 5) was determined by comparing the response of extracted analyte in spiked blank matrix with the response of analyte spiked after the extraction of blank matrix. The matrix effect (in percent*, n* = 5) was determined by comparing the response of analyte spiked after the extraction of blank matrix with the response of analyte in neat solution. Matrix effect was calculated using the equation described by Chambers et al. [64].

## 5. Conclusions

The presented method is fast, simple, and sensitive due to the use of a mass spectrometer in MRM mode. The method was validated in accordance with SWGTOX guidelines. The method’s limit of quantification (LOQ) was in the range of 0.1 to 0.5 ng/mL. All compounds had a coefficient of determination (R²) above 0.995. Intraday and interday precision (RSD%) and accuracy (RE%) for low and high QC levels were within 1.7–12.3% and −14.4 to 14.1%, respectively. Very good recovery (80.0–119.6%) and matrix effect (±20.0%) values were achieved for all compounds. Fragmentation spectra were collected for all substances tested, and high-resolution spectra were presented for landiolol and metipranolol, as they are not available in commercial HRMS databases. The developed method was verified and applied to authentic postmortem samples. After verification and revalidation, this method can be easily adapted for use in clinical and forensic toxicology laboratories, as well as for therapeutic drug monitoring (TDM).

## Figures and Tables

**Table 1:** Multiple reaction monitoring (MRM) conditions used in the UHPLC-QqQ-MS/MS method for determination of β-blockers in biological samples.

No.	Substance	Precursor Ion (*m*/*z*)	Product Ion (*m*/*z*)	Dwell Time (ms)	Q1 Pre-Bias (V)	Collision Energy (V)	Q3 Pre-Bias (V)	Retention Time (min)
1	Sotalol	273.2	255.2 *	15	−10	−13	−17	2.42
			213.15	15	−14	−19	−22	
			133.25	15	−15	−26	−24	
2	Atenolol	267.0	145.2 *	13	−16	−24	−15	2.45
			190.15	13	−14	−21	−21	
			225.2	13	−14	−18	−23	
3	Carteolol	293.0	237.15 *	8	−15	−17	−26	3.22
			202.1	8	−15	−21	−21	
			164.1	8	−15	−23	−29	
4	Pindolol	249.0	116.2 *	15	−14	−17	−18	3.39
			172.2	15	−27	−19	−16	
			74.25	15	−26	−26	−11	
5	Timolol	317.0	261.05 *	8	−16	−20	−23	3.98
			74.15	8	−15	−25	−24	
			244.2	8	−15	−23	−25	
6	Acebutolol	337.2	116.2 *	6	−10	−22	−21	4.02
			319.3	6	−18	−17	−20	
			260.1	6	−19	−19	−29	
7	Metoprolol	268.0	116.2 *	8	−13	−20	−11	4.14
			98.05	8	−13	−21	−15	
			74.15	8	−13	−22	−30	
8	Esmolol	296.0	145.2 *	4	−16	−26	−14	4.45
			219.2	4	−12	−21	−24	
			254.15	4	−22	−19	−17	
9	Celiprolol	380.4	251.2 *	4	−21	−23	−15	4.55
			307.2	4	−26	−18	−14	
			324.3	4	−20	−19	−21	
10	Oxprenolol	266.0	72.3 *	4	−14	−22	−13	4.70
			225.2	4	−15	−14	−27	
			116.2	4	−14	−17	−20	
11	Bisoprolol	326.0	116 *	6	−20	−20	−20	4.95
			74.15	6	−15	−25	−26	
			56.1	6	−15	−35	−21	
12	Landiolol	510.0	157.25 *	4	−26	−38	−29	5.04
			423.25	4	−26	−21	−20	
			365.15	4	−26	−24	−25	
13	Metipranolol	310.0	191.1 *	4	−12	−25	−30	5.19
			233.2	4	−12	−19	−26	
			165.2	4	−17	−30	−28	
14	Propranolol	260.0	116.1 *	6	−11	−20	−17	5.25
			183.15	6	−11	−18	−18	
			56.1	6	−11	−29	−23	
15	Alprenolol	250.3	116.25 *	4	−13	−17	−11	5.33
			91.05	4	−13	−42	−16	
			173.25	4	−13	−16	−20	
16	Betaxolol	308.1	116.15 *	6	−15	−21	−11	5.46
			72.05	6	−14	−24	−27	
			98.15	6	−15	−23	−14	
17	Carverdilol	407.0	100.2 *	8	−10	−30	−10	6.07
			194.2	8	−23	−37	−20	
			283.2	8	−21	−22	−30	
18	Nebivolol	406.2	151.25 *	21	−12	−31	−15	6.42
			103.2	21	−12	−54	−18	
			123.25	21	−21	−41	−12	
IS1	Atenolol-d_7_	274.1	145.15 *	13	−14	−28	−24	2.44
			190.25	13	−15	−21	−19	
			226.1	13	−14	−18	−25	
IS2	Metoprolol-d_7_	275.1	123.35 *	6	−14	−21	−24	4.13
			191.2	6	−19	−20	−19	
			159.15	6	−10	−23	−17	
IS3	Propranolol-d_7_	267.0	189.3	4	−14	−19	−12	5.22
			116.25 *	4	−17	−21	−20	
			98.15	4	−10	−21	−15	

*—ions selected for quantitative analysis.

**Table 2:** Comparison of the liquid chromatography–mass spectrometry methods for determination of β-blockers in biological samples.

Biological Sample (Volume)	Number of Determined β-Blockers	Internal Standard	Sample Preparation Technique	Method	The Lowest LOQ of Method [ng/mL]	Year	Reference
Serum (1 mL)	6	Metoprolol-d_7_	LLE (pH9, butyl acetate)	UHPLC-TOF (ESI, Scan)	0.3	2017	[39]
Biological fluids (2 mL) Solid tissues (200 mg) ^a^	3	Clenbuterol	LLE supported by Extrelut^®^ columns	HPLC-MS (APCI, SIM)	10	2004	[48]
Urine (5 mL)	32	Bupranolol	Enzymatic hydrolysis with β-glucuronidase followed by LLE	HPLC-MS/MS (APCI, MRM)	–	2001	[40]
Plasma (1 mL)	4	Pindolol	Dilution and filtration	LC-MS/MS (ESI, SRM)	10	2008	[41]
Plasma (1 mL)	5	–	SPE with derivatization ^b^	LC-MS/MS (ESI, MRM)	0.1	2020	[42]
Plasma (500 µL)	22	Trimipramine-d_3_	SPE	LC-MS (APCI, SIM)	2.5	2004	[43]
Urine (2 mL)	19	Furosemide-d_5_ Benzoylecgonine-d_3_	SPE	UPLC-MS/MS (ESI, MRM)	–	2009	[44]
Serum or urine (200 µL)	6	Sulfapyridine	Fast fabric phase sorptive extraction	UHPLC-MS/MS (ESI, MRM)	2	2021	[45]
Biological fluids (100 µL) Solid tissues (100 mg)	18	Atenolol-d_7_ Metorpolol-d_7_ Propranolol-d_7_	LLE (pH9, ethyl acetate)	UHPLC-MS/Ms (ESI, MRM)	0.1	2024	Presented method

^a^ Analysed following biological samples: blood, heart, liver, lung, vitreous humour, kidney, brain, urine, and gastric liquid—information was not provided; ^b^ derivatisation with hydrazonoyl chloride (UOSA54) and dansyl chloride (DNS).

## Data Availability

The original contributions presented in the study are included in the article/Supplementary Materials, further inquiries can be directed to the corresponding author/s.

## Supplementary Materials

The following supporting information can be downloaded at: https://www.mdpi.com/article/10.3390/molecules29194585/s1, Table S1: Validation results; Figure S1: Fragmentation spectra (QqQ) and MRM chromatograms.
