# Supplementary material for: Application of UHPLC-QqQ-MS/MS Method for Quantification of Beta-Adrenergic Blocking Agents (β-Blockers) in Human Postmortem Specimens
Source: Molecules. 2024 Sep 27;29(19):4585. doi: 10.3390/molecules29194585 (PMC11477679; doi:10.3390/molecules29194585)

Table S1. Validation results.

	Substance	The coefficient of determination (R^2)	Internal standard	Range of calibration curve [ng/mL]	Concentration level [ng/mL]	Precision [%]*	Accuracy [%]*	Recovery [%]*	Matrix effect [%]*
1.	Sotalol	0.998	IS-2	0.5 – 1000	5<br>500	3.5<br>3.8	-3.9<br>-10.5	80.0<br>83.7	-20.0<br>-16.3
2.	Atenolol	0.999	IS-1	0.5 – 1000	5<br>500	2.1<br>3.1	0.0<br>-6.9	114.9<br>83.5	14.9<br>-16.5
3.	Carteolol	0.999	IS-2	0.5 – 200	5<br>200	5.3<br>5.2	-6.9<br>13.1	90.0<br>90.8	-10.0<br>-9.2
4.	Pindolol	0.998	IS-3	0.1 – 200	5<br>200	4.4<br>5.3	8.6<br>9.3	119.6<br>106.7	19.6<br>6.7
5.	Timolol	0.998	IS-3	0.1 – 200	5<br>200	4.8<br>1.7	13.3<br>11.0	97.3<br>105.7	-2.7<br>5.7
6.	Acebutolol	0.999	IS-2	0.1 – 200	5<br>200	5.4<br>3.2	-8.7<br>7.4	93.4<br>88.7	-6.6<br>-11.3
7.	Metoprolol	0.999	IS-2	0.2 – 1000	5<br>500	3.9<br>3.4	0.1<br>-7.8	92.1<br>80.7	-7.9<br>-19.3
8.	Esmolol	0.998	IS-2	0.2 – 500	5<br>500	3.7<br>3.2	14.1<br>3.6	99.7<br>81.1	-0.3<br>-18.9
9.	Celiprolol	0.999	IS-2	0.2 – 1000	5<br>500	12.3<br>9.3	-14.4<br>-11.5	104.4<br>94.9	4.4<br>-5.1
10.	Oxprenolol	0.999	IS-2	0.2 – 200	5<br>200	10.9<br>4.0	-12.4<br>1.4	100.2<br>97.3	0.2<br>-2.7
11.	Bisoprolol	0.999	IS-3	0.1 – 200	5<br>200	4.7<br>12.3	-6.3<br>10.8	102.2<br>105.1	2.2<br>5.1
12.	Landiolol	0.996	IS-3	0.5 – 1000	5<br>500	4.5<br>3.0	-3.0<br>-12.6	87.3<br>104.0	-12.7<br>4.0
13.	Metipranolol	0.999	IS-3	0.5 – 1000	5<br>500	4.3<br>4.6	-12.3<br>-1.4	81.2<br>101.2	-18.8<br>1.2
14.	Propranolol	0.999	IS-3	0.2 – 1000	5<br>500	4.1<br>1.7	3.1<br>-14.1	96.9<br>104.2	-3.1<br>4.2
15.	Alprenolol	0.999	IS-3	0.2 – 1000	5<br>500	6.8<br>3.8	8.8<br>-0.9	96.6<br>106.1	-3.4<br>6.1
16.	Betaxolol	0.999	IS-3	0.5 – 500	5<br>500	5.1<br>3.6	12.6<br>-1.0	97.3<br>104.7	-2.7<br>4.7
17.	Carverdilol	0.999	IS-3	0.2 – 500	5<br>500	4.0<br>5.1	6.6<br>-5.4	91.8<br>107.9	-8.2<br>7.9
18.	Nebivolol	0.999	IS-3	0.2 – 500	5<br>500	4.0<br>5.1	-7.0<br>-4.2	88.4<br>100.8	-11.6<br>-0.8

IS-1: Atenolol-d_7
IS-2: Metoprolol-d_7
IS-3: Propranolol-d_7
* n=5

Figure S1

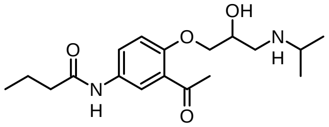

Acebutolol

Molecular Formula: $C_{18}H_{28}N_2O_4$

Formula Weight: 336.432 Da

[M+H]$^+$: 337.212184 m/z

Acebutolol

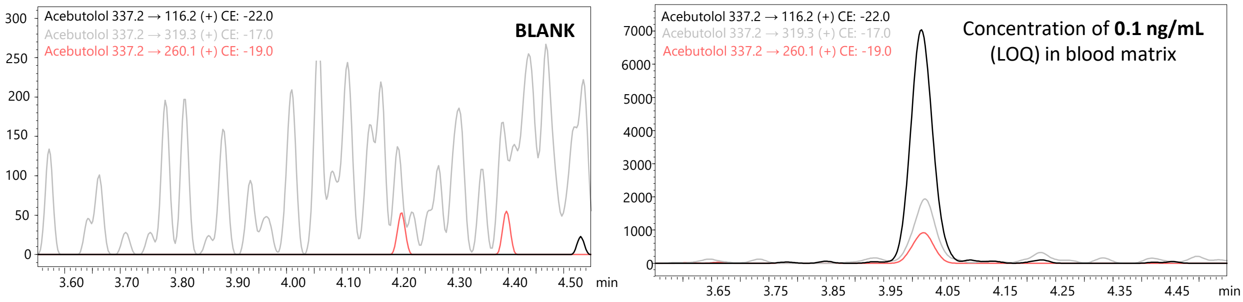

QqQ-MS/MS: Detection of the investigated compounds was achieved with the use of a triple quadrupole mass spectrometer (QqQ, Shimadzu 8050, Kyoto, Japan) in positive mode. The spectrometer was equipped with an electrospray ionization (ESI) source. Fragmentation MS/MS spectra were acquired by conducting a product ion scan experiment at five collision energies CE (**A:** –5, **B:** –10, **C:** –20, **D:** –35, **E:** –40, and **F:** –50 V).

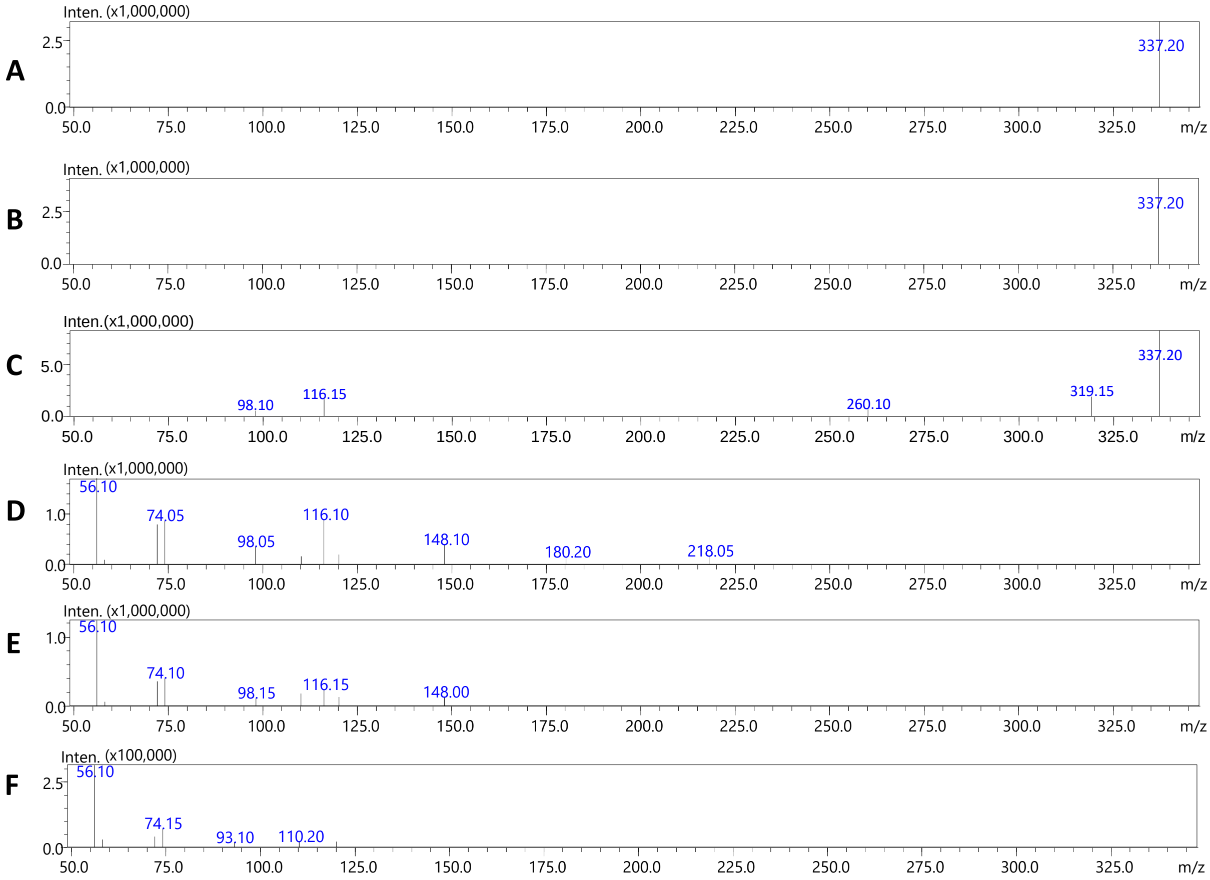

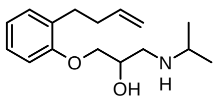

Alprenolol

Molecular Formula: $C_{15}H_{23}NO_2$

Formula Weight: 249.354 Da

$[M+H]^+$: 250.180155 m/z

Alprenolol

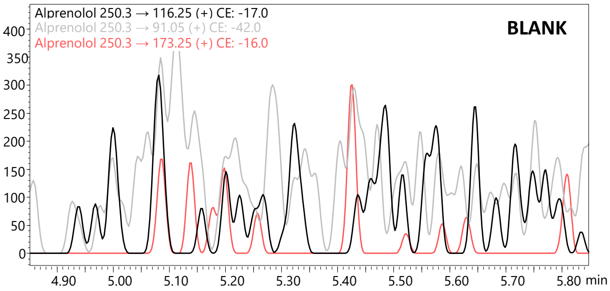

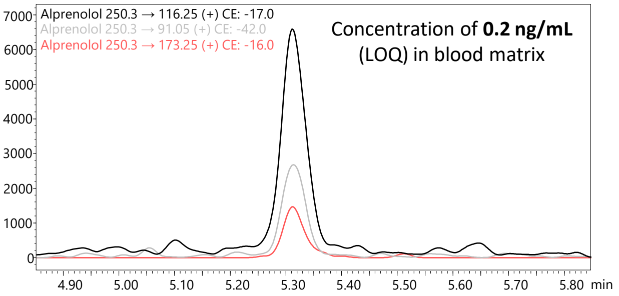

QqQ-MS/MS: Detection of the investigated compounds was achieved with the use of a triple quadrupole mass spectrometer (QqQ, Shimadzu 8050, Kyoto, Japan) in positive mode. The spectrometer was equipped with an electrospray ionization (ESI) source. Fragmentation MS/MS spectra were acquired by conducting a product ion scan experiment at five collision energies CE (**A:** –5, **B:** –10, **C:** –20, **D:** –35, **E:** –40, and **F:** –50 V).

A

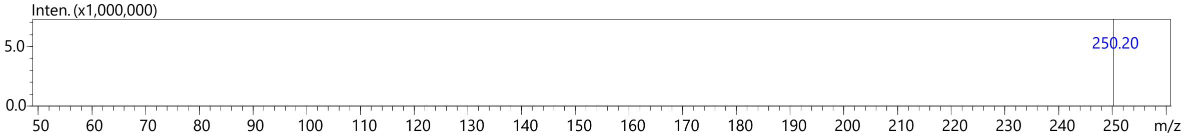

B

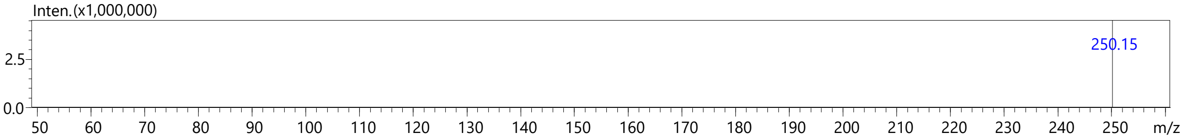

C

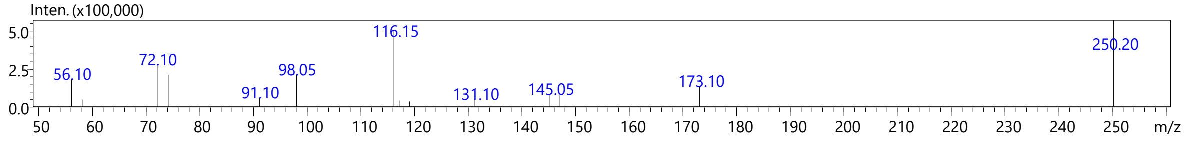

D

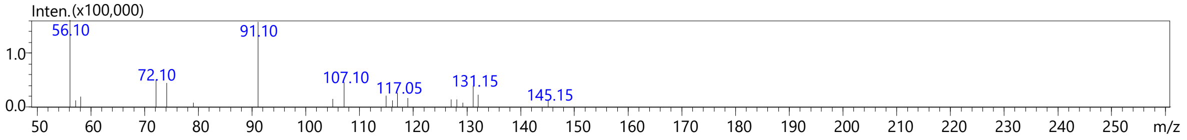

E

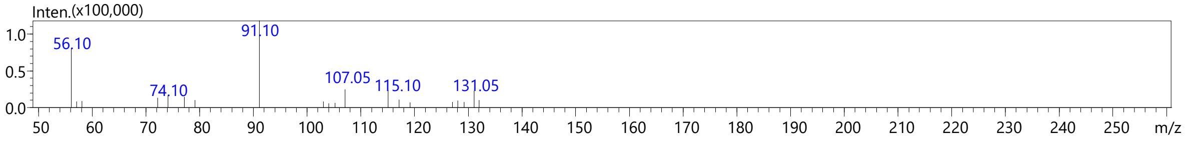

F

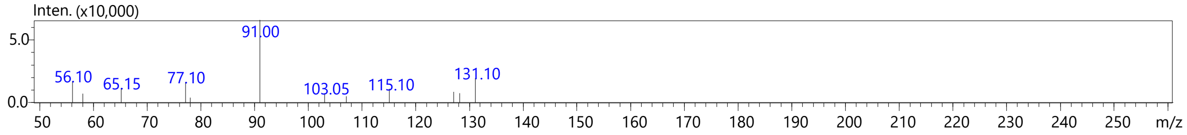

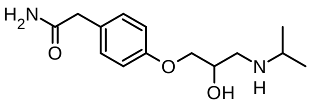

Atenolol

Molecular Formula: $C_{14}H_{22}N_2O_3$

Formula Weight: 266.341 Da

[M+H]$^{+}$: 267.170319 m/z

Atenolol

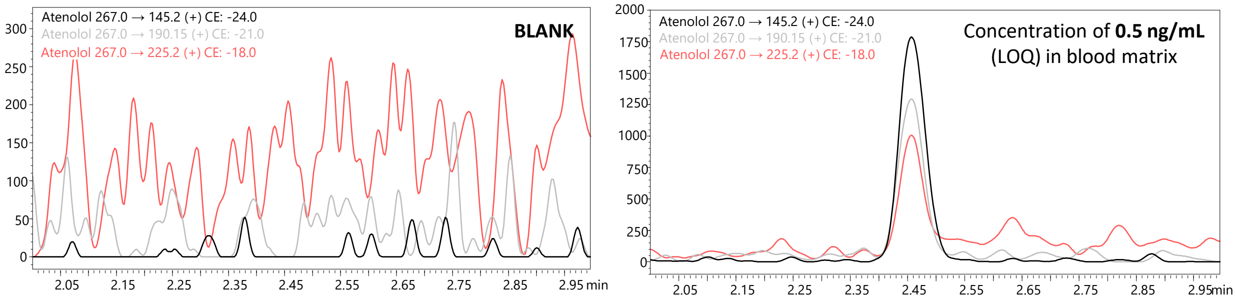

QqQ-MS/MS: Detection of the investigated compounds was achieved with the use of a triple quadrupole mass spectrometer (QqQ, Shimadzu 8050, Kyoto, Japan) in positive mode. The spectrometer was equipped with an electrospray ionization (ESI) source. Fragmentation MS/MS spectra were acquired by conducting a product ion scan experiment at five collision energies CE (**A:** –5, **B:** –10, **C:** –20, **D:** –35, **E:** –40, and **F:** –50 V).

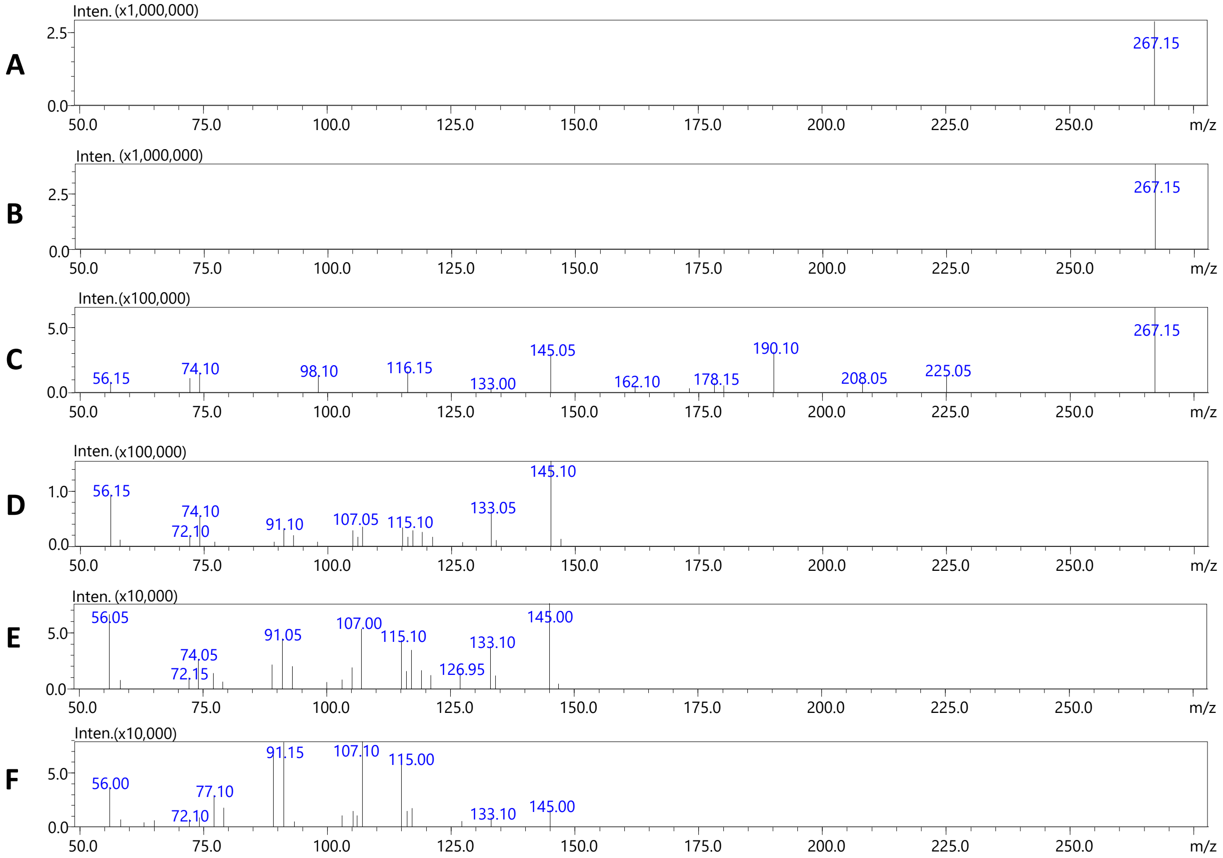

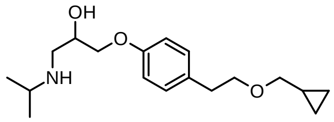

Betaxolol

Molecular Formula: $C_{18}H_{29}NO_3$

Formula Weight: 307.434 Da

$[M+H]^+$: 308.22202 m/z

Betaxolol

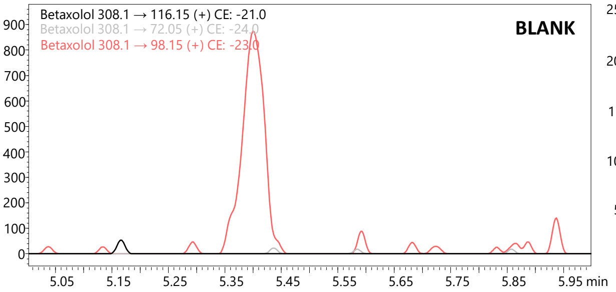

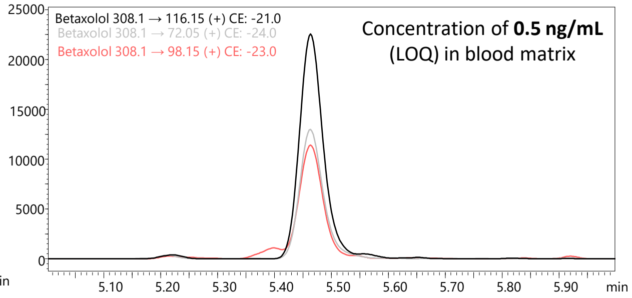

QqQ-MS/MS: Detection of the investigated compounds was achieved with the use of a triple quadrupole mass spectrometer (QqQ, Shimadzu 8050, Kyoto, Japan) in positive mode. The spectrometer was equipped with an electrospray ionization (ESI) source. Fragmentation MS/MS spectra were acquired by conducting a product ion scan experiment at five collision energies CE (**A:** –5, **B:** –10, **C:** –20, **D:** –35, **E:** –40, and **F:** –50 V).

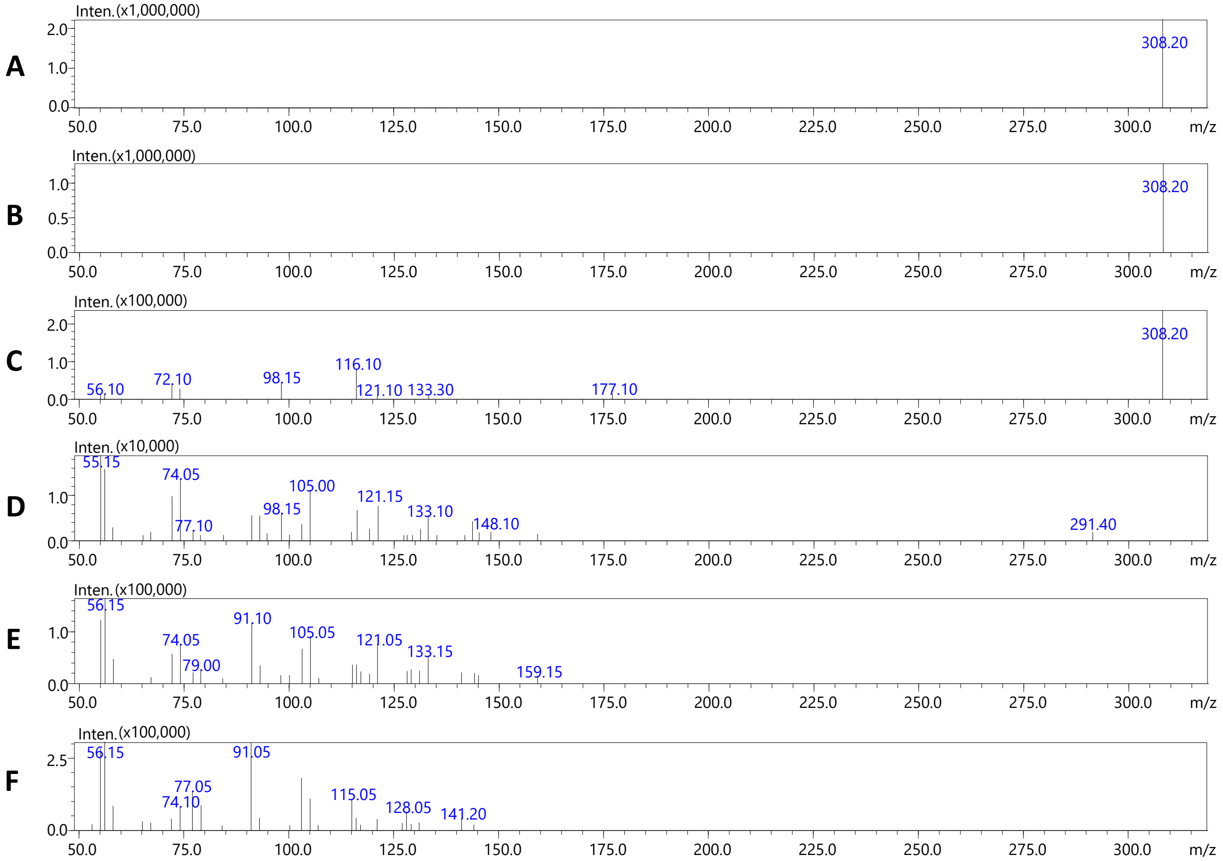

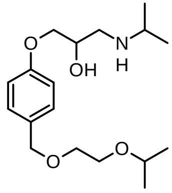

Bisoprolol

Molecular Formula: $C_{18}H_{31}NO_4$

Formula Weight: 325.449 Da

[M+H]$^+$: 326.232585 m/z

Bisoprolol

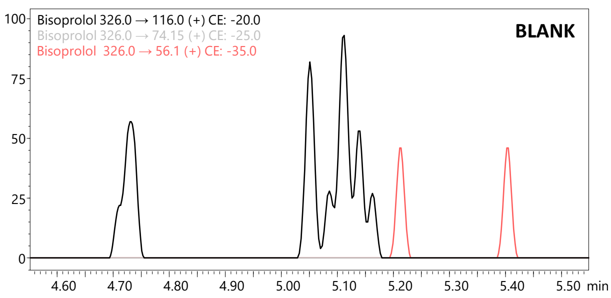

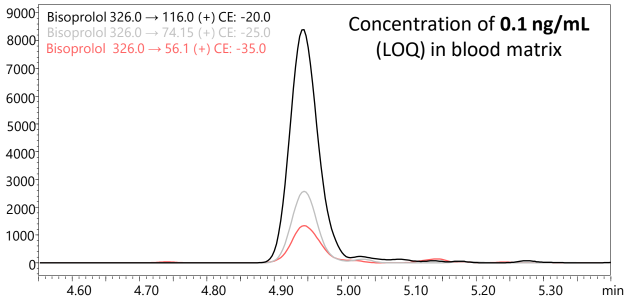

QqQ-MS/MS: Detection of the investigated compounds was achieved with the use of a triple quadrupole mass spectrometer (QqQ, Shimadzu 8050, Kyoto, Japan) in positive mode. The spectrometer was equipped with an electrospray ionization (ESI) source. Fragmentation MS/MS spectra were acquired by conducting a product ion scan experiment at five collision energies CE (**A:** –5, **B:** –10, **C:** –20, **D:** –35, **E:** –40, and **F:** –50 V).

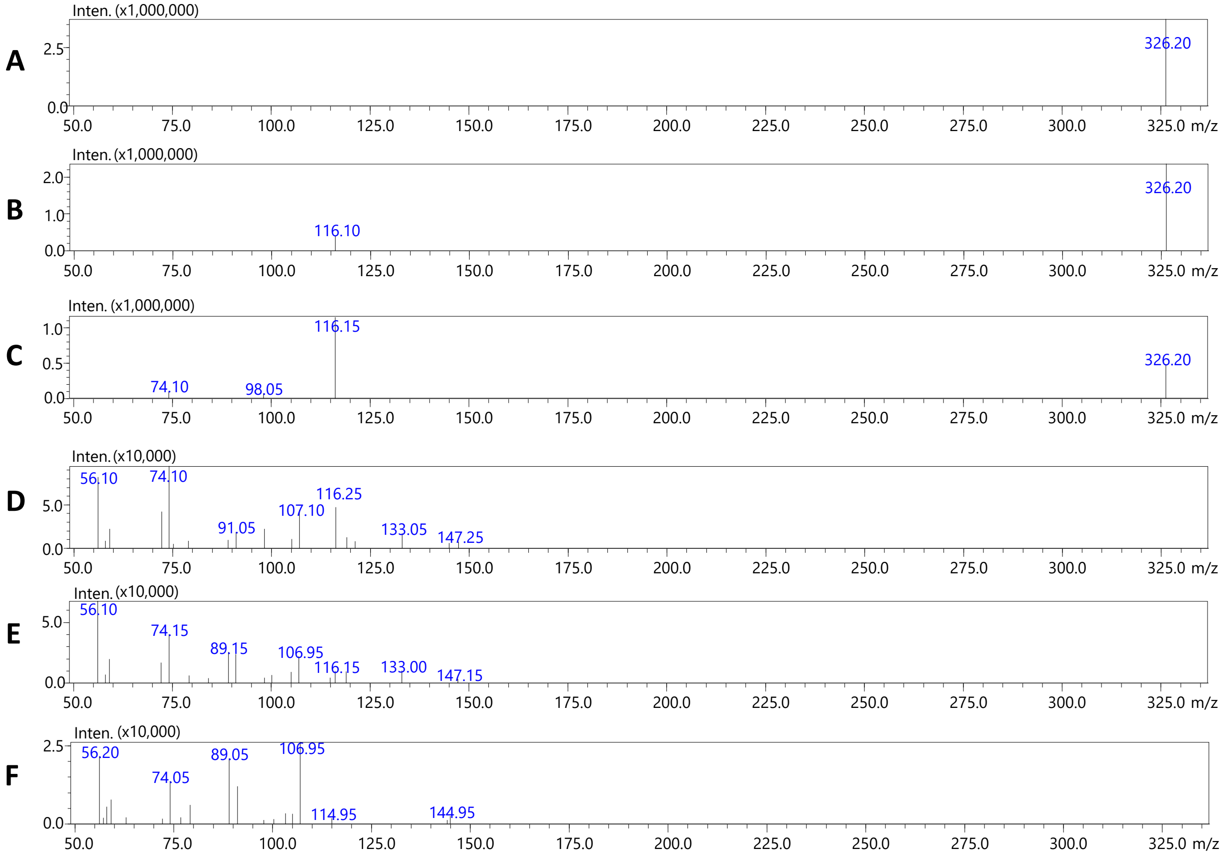

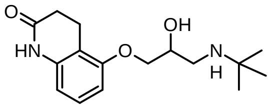

Carteolol

Molecular Formula: $C_{16}H_{24}N_2O_3$

Formula Weight: 292.379 Da

[M+H]$^+$: 293.185969 m/z

Carteolol

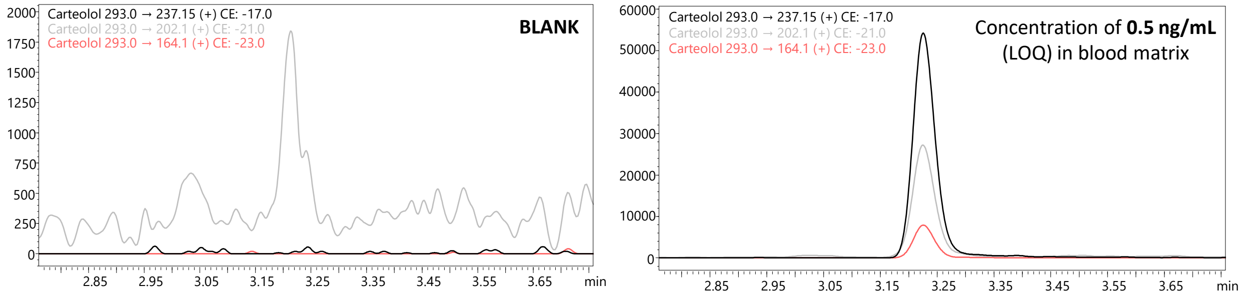

QqQ-MS/MS: Detection of the investigated compounds was achieved with the use of a triple quadrupole mass spectrometer (QqQ, Shimadzu 8050, Kyoto, Japan) in positive mode. The spectrometer was equipped with an electrospray ionization (ESI) source. Fragmentation MS/MS spectra were acquired by conducting a product ion scan experiment at five collision energies CE (**A:** –5, **B:** –10, **C:** –20, **D:** –35, **E:** –40, and **F:** –50 V).

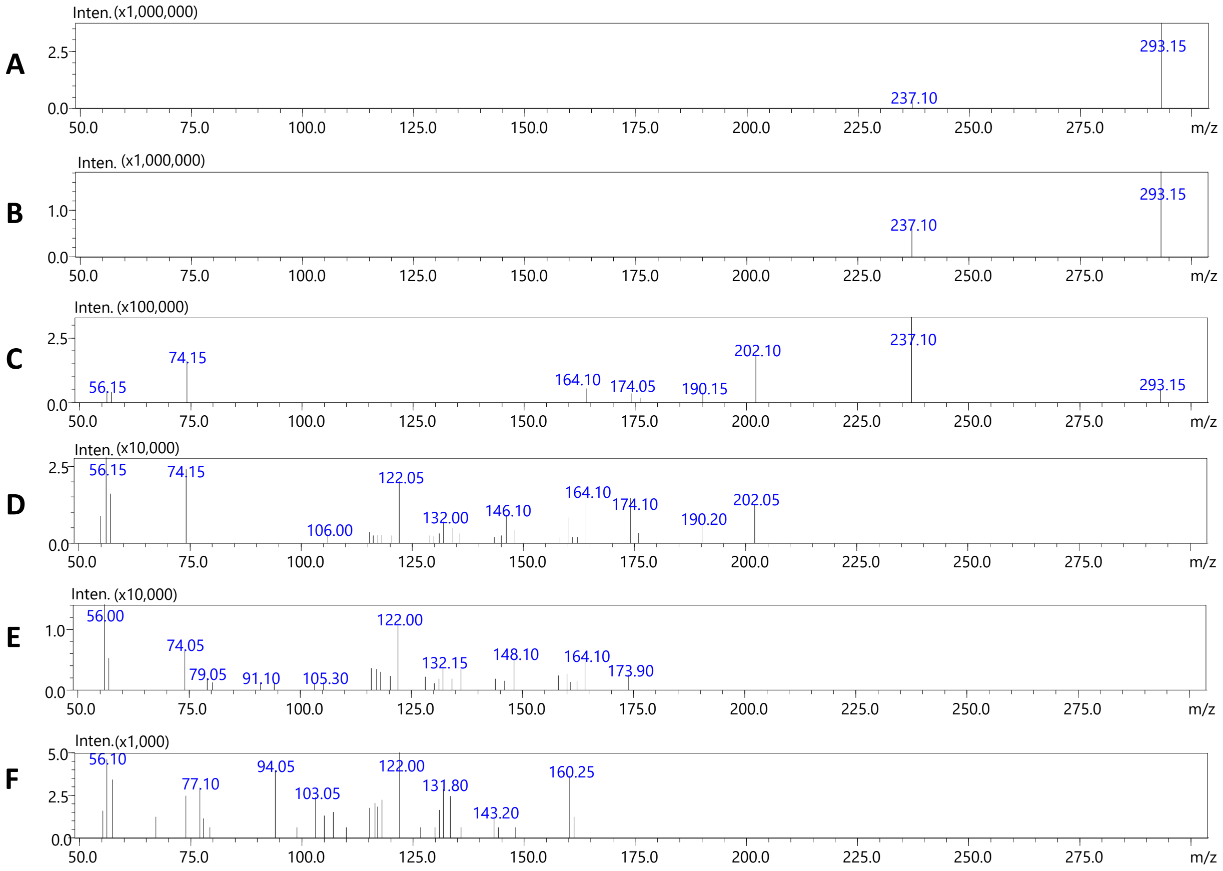

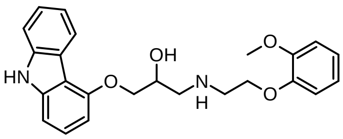

Carverdilol

Molecular Formula: $C_{24}H_{26}N_2O_4$

Formula Weight: 406.482 Da

[M+H]$^+$: 407.196534 m/z

Carverdilol

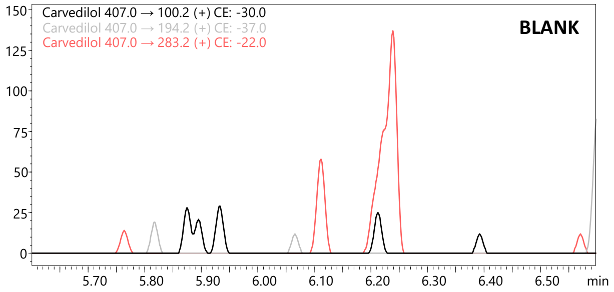

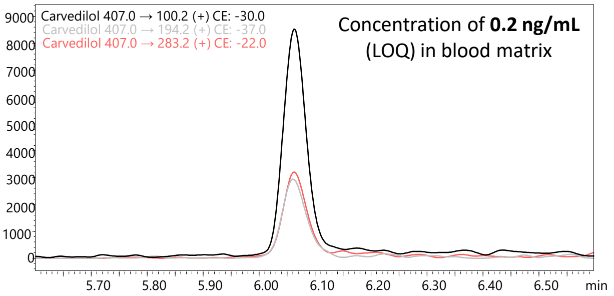

QqQ-MS/MS: Detection of the investigated compounds was achieved with the use of a triple quadrupole mass spectrometer (QqQ, Shimadzu 8050, Kyoto, Japan) in positive mode. The spectrometer was equipped with an electrospray ionization (ESI) source. Fragmentation MS/MS spectra were acquired by conducting a product ion scan experiment at five collision energies CE (**A:** –5, **B:** –10, **C:** –20, **D:** –35, **E:** –40, and **F:** –50 V).

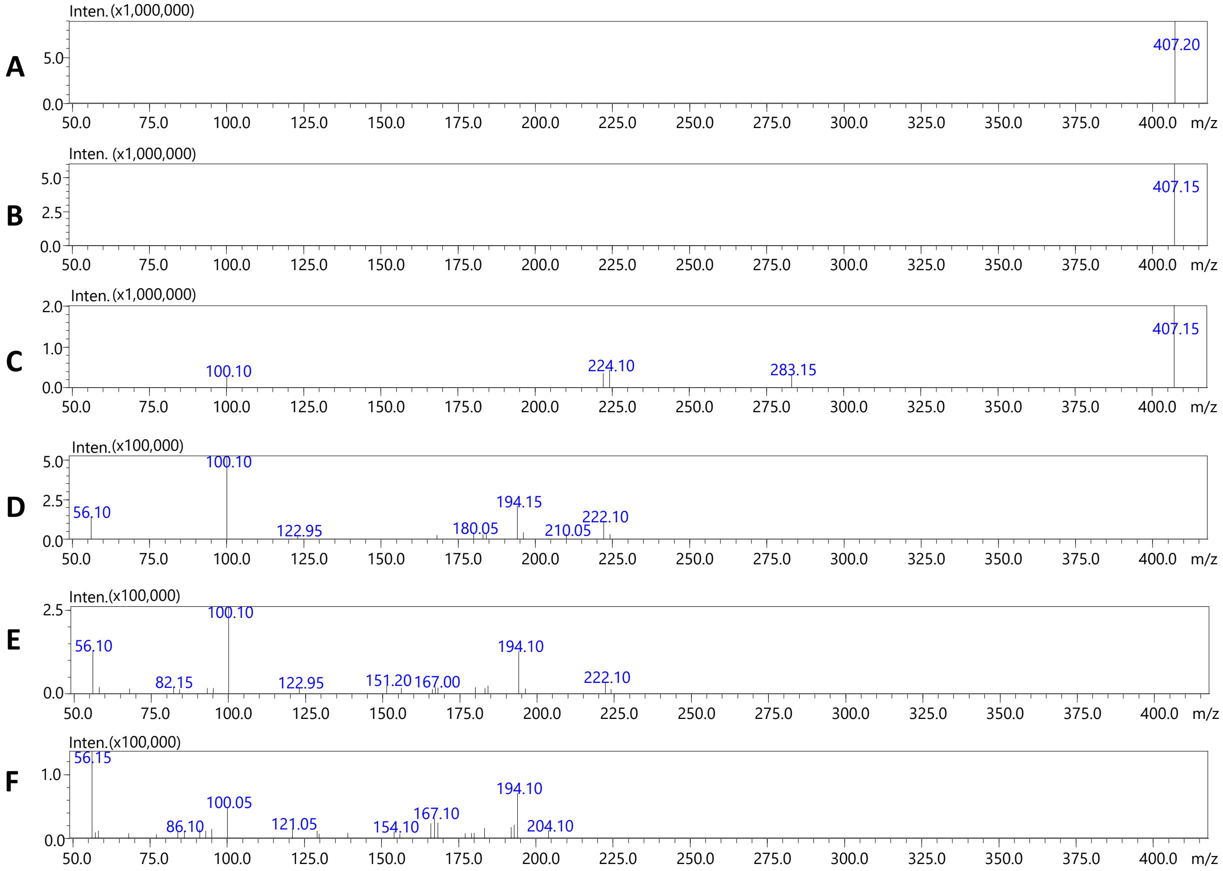

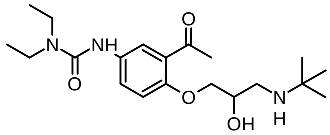

Celiprolol

Molecular Formula: $C_{20}H_{33}N_3O_4$

Formula Weight: 379.501 Da

[M+H]$^+$: 380.254383 m/z

Celiprolol

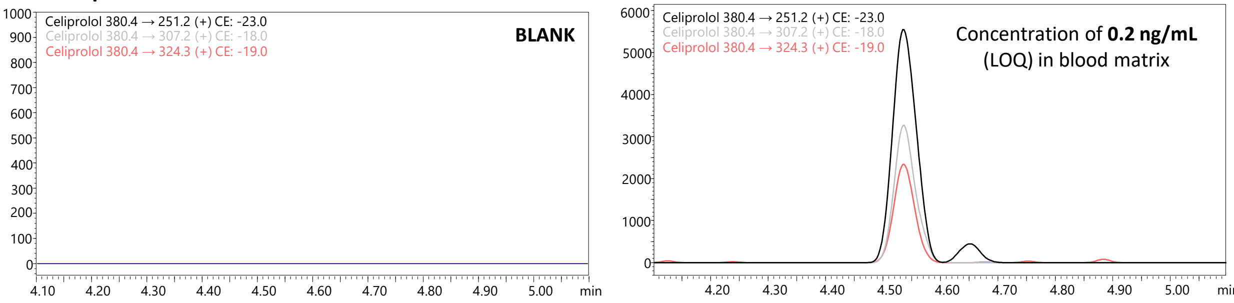

QqQ-MS/MS: Detection of the investigated compounds was achieved with the use of a triple quadrupole mass spectrometer (QqQ, Shimadzu 8050, Kyoto, Japan) in positive mode. The spectrometer was equipped with an electrospray ionization (ESI) source. Fragmentation MS/MS spectra were acquired by conducting a product ion scan experiment at five collision energies CE (**A:** –5, **B:** –10, **C:** –20, **D:** –35, **E:** –40, and **F:** –50 V).

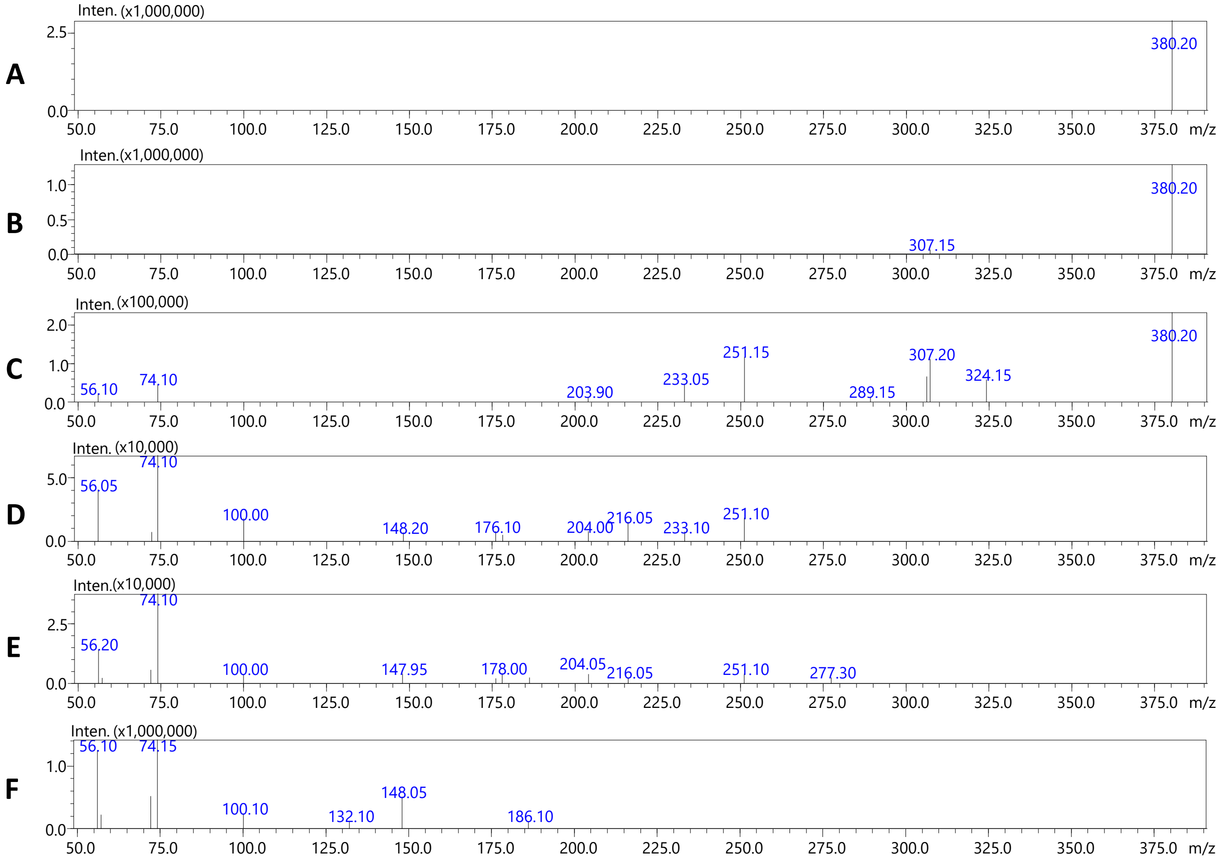

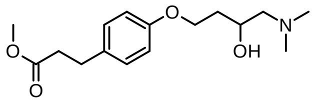

Esmolol

Molecular Formula: $C_{16}H_{25}NO_4$

Formula Weight: 295.379 Da

[M+H]⁺: 296.185635 m/z

Esmolol

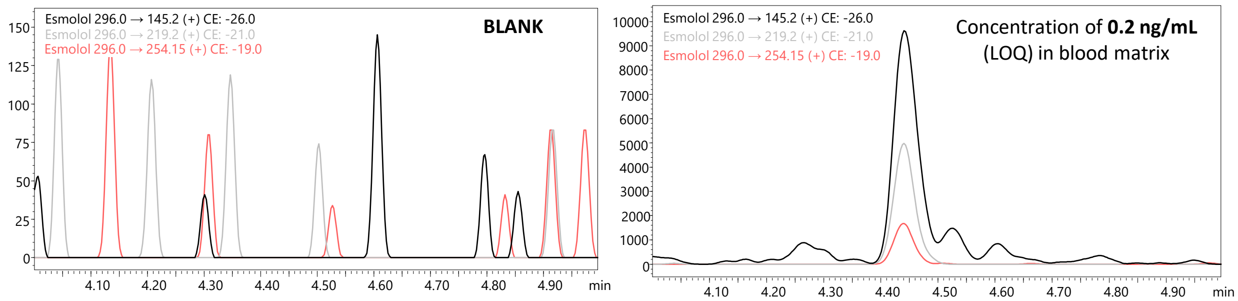

QqQ-MS/MS: Detection of the investigated compounds was achieved with the use of a triple quadrupole mass spectrometer (QqQ, Shimadzu 8050, Kyoto, Japan) in positive mode. The spectrometer was equipped with an electrospray ionization (ESI) source. Fragmentation MS/MS spectra were acquired by conducting a product ion scan experiment at five collision energies CE (**A:** –5, **B:** –10, **C:** –20, **D:** –35, **E:** –40, and **F:** –50 V).

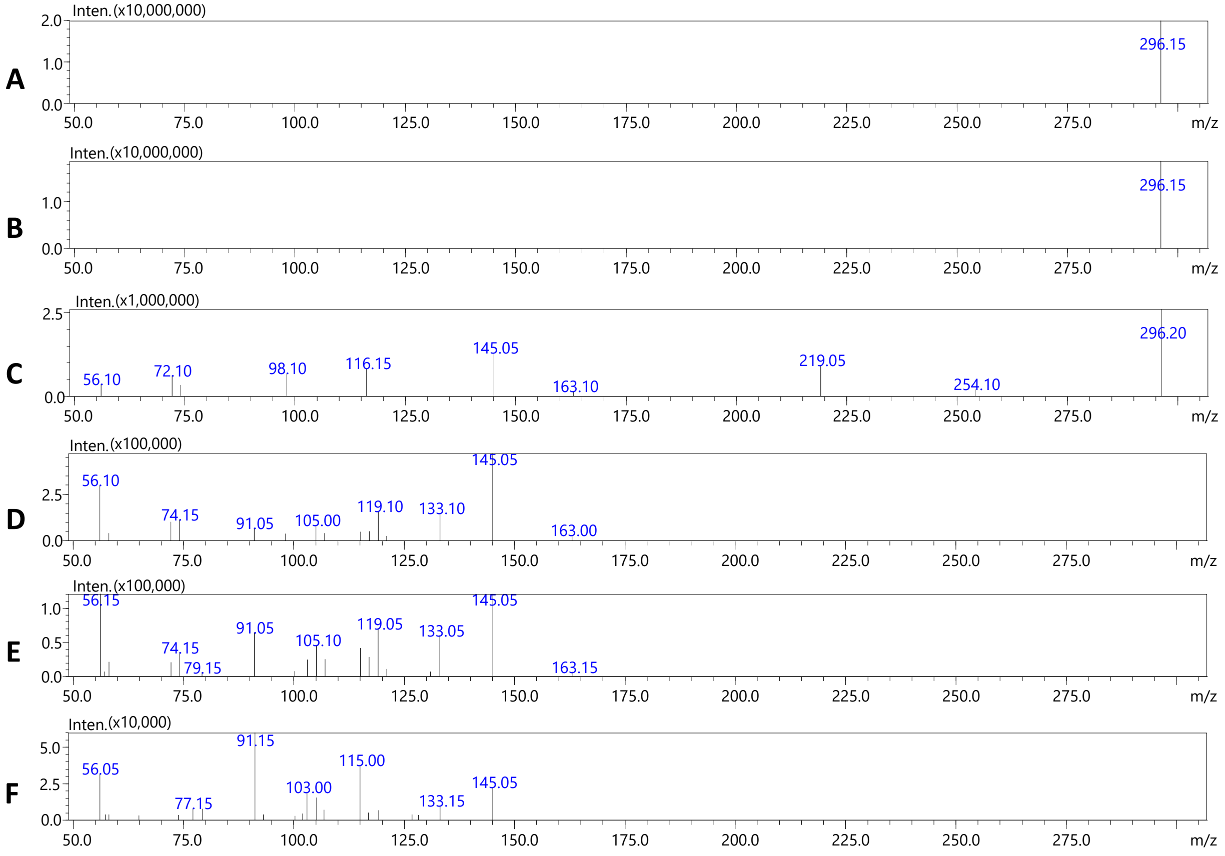

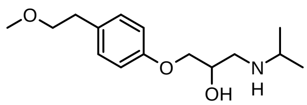

Metoprolol

Molecular Formula: $C_{15}H_{25}NO_3$

Formula Weight: 267.369 Da

[M+H]$^+$: 268.19072 m/z

Metoprolol

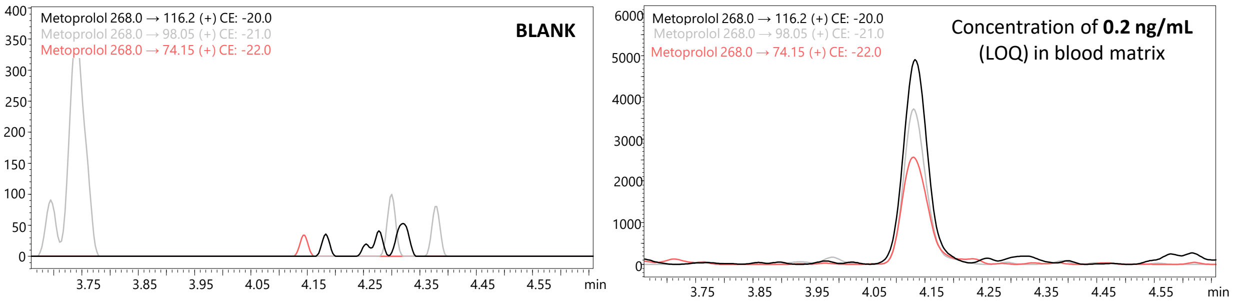

QqQ-MS/MS: Detection of the investigated compounds was achieved with the use of a triple quadrupole mass spectrometer (QqQ, Shimadzu 8050, Kyoto, Japan) in positive mode. The spectrometer was equipped with an electrospray ionization (ESI) source. Fragmentation MS/MS spectra were acquired by conducting a product ion scan experiment at five collision energies CE (**A:** –5, **B:** –10, **C:** –20, **D:** –35, **E:** –40, and **F:** –50 V).

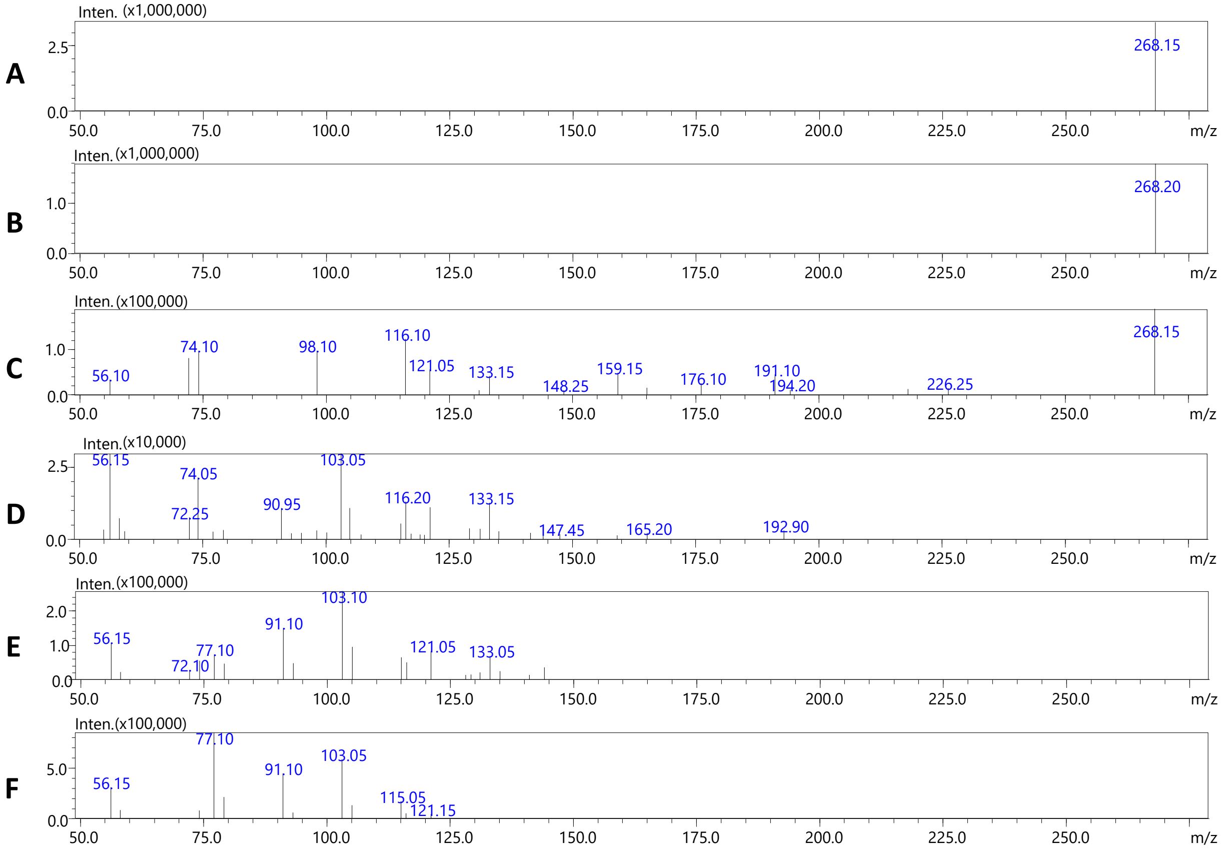

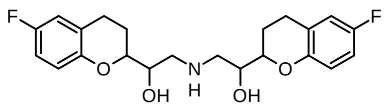

Nebivolol

Molecular Formula: $C_{22}H_{25}F_2NO_4$

Formula Weight: 405.442 Da

[M+H]$^+$: 406.182441 m/z

Nebivolol

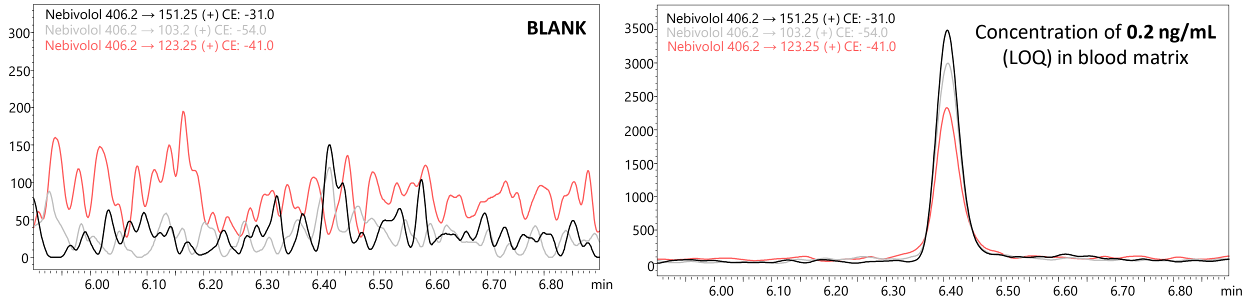

QqQ-MS/MS: Detection of the investigated compounds was achieved with the use of a triple quadrupole mass spectrometer (QqQ, Shimadzu 8050, Kyoto, Japan) in positive mode. The spectrometer was equipped with an electrospray ionization (ESI) source. Fragmentation MS/MS spectra were acquired by conducting a product ion scan experiment at five collision energies CE (**A:** –5, **B:** –10, **C:** –20, **D:** –35, **E:** –40, and **F:** –50 V).

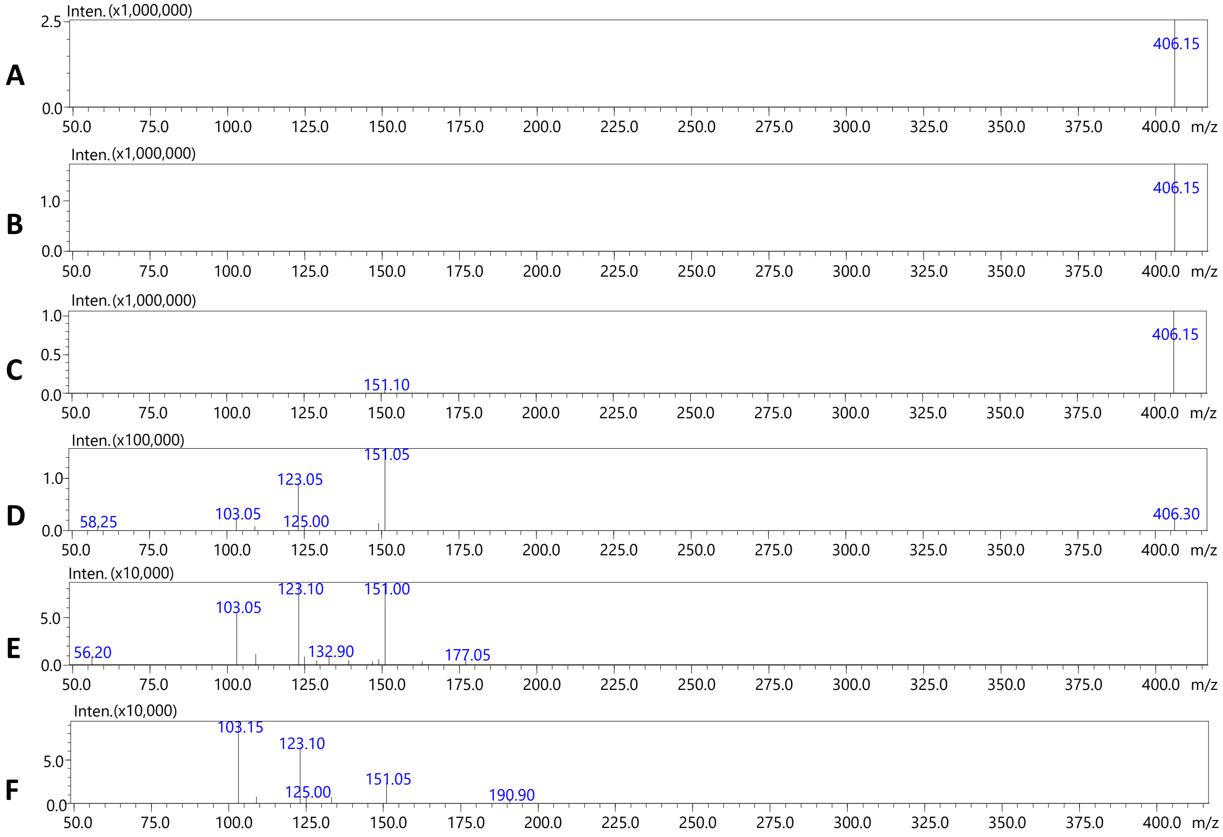

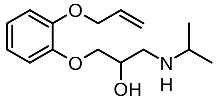

Oxprenolol

Molecular Formula: $C_{15}H_{23}NO_3$

Formula Weight: 265.353 Da

$[M+H]^+$: 266.17507 m/z

Oxprenolol

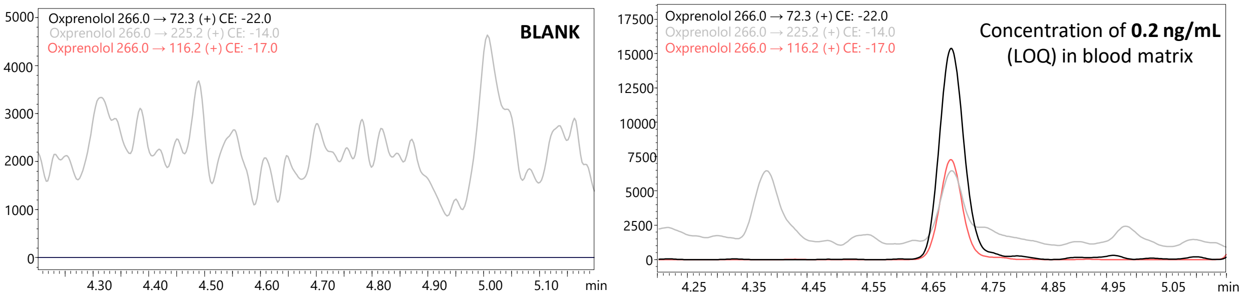

QqQ-MS/MS: Detection of the investigated compounds was achieved with the use of a triple quadrupole mass spectrometer (QqQ, Shimadzu 8050, Kyoto, Japan) in positive mode. The spectrometer was equipped with an electrospray ionization (ESI) source. Fragmentation MS/MS spectra were acquired by conducting a product ion scan experiment at five collision energies CE (**A:** –5, **B:** –10, **C:** –20, **D:** –35, **E:** –40, and **F:** –50 V).

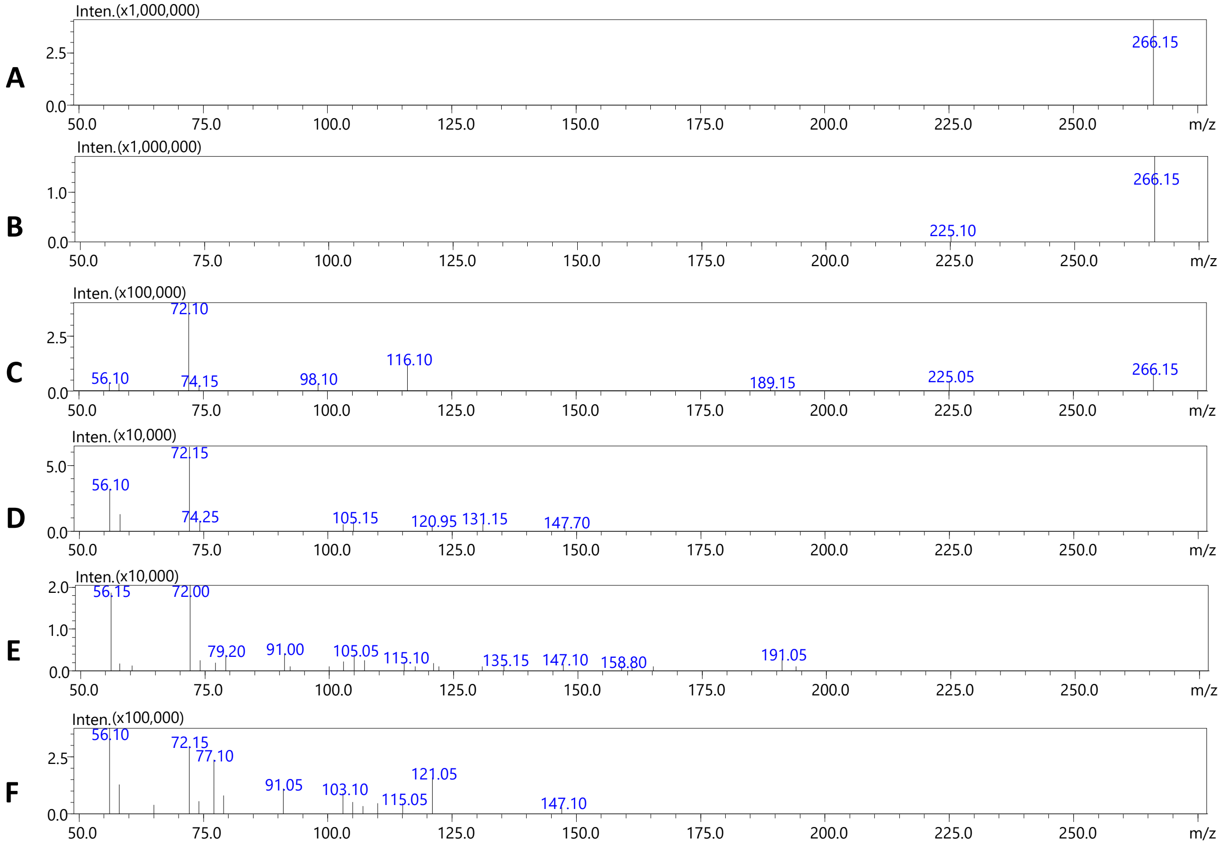

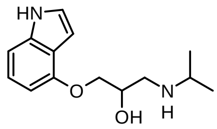

Pindolol

Molecular Formula: $C_{14}H_{20}N_2O_2$

Formula Weight: 248.326 Da

$[M+H]^+$: 249.159754 m/z

Pindolol

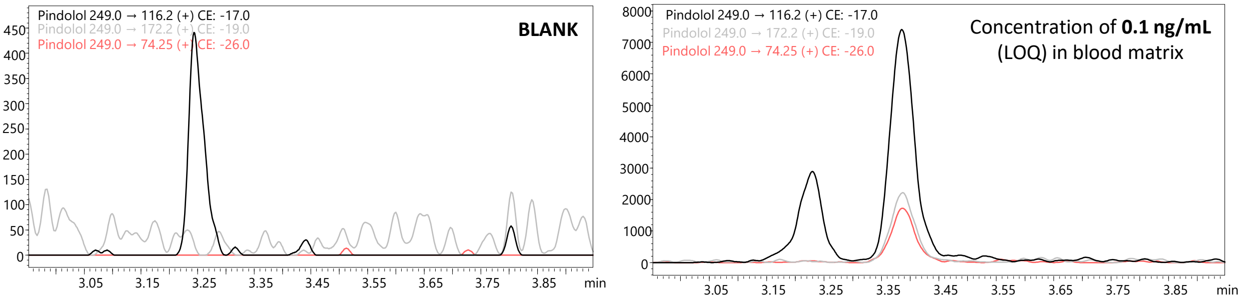

QqQ-MS/MS: Detection of the investigated compounds was achieved with the use of a triple quadrupole mass spectrometer (QqQ, Shimadzu 8050, Kyoto, Japan) in positive mode. The spectrometer was equipped with an electrospray ionization (ESI) source. Fragmentation MS/MS spectra were acquired by conducting a product ion scan experiment at five collision energies CE (**A:** –5, **B:** –10, **C:** –20, **D:** –35, **E:** –40, and **F:** –50 V).

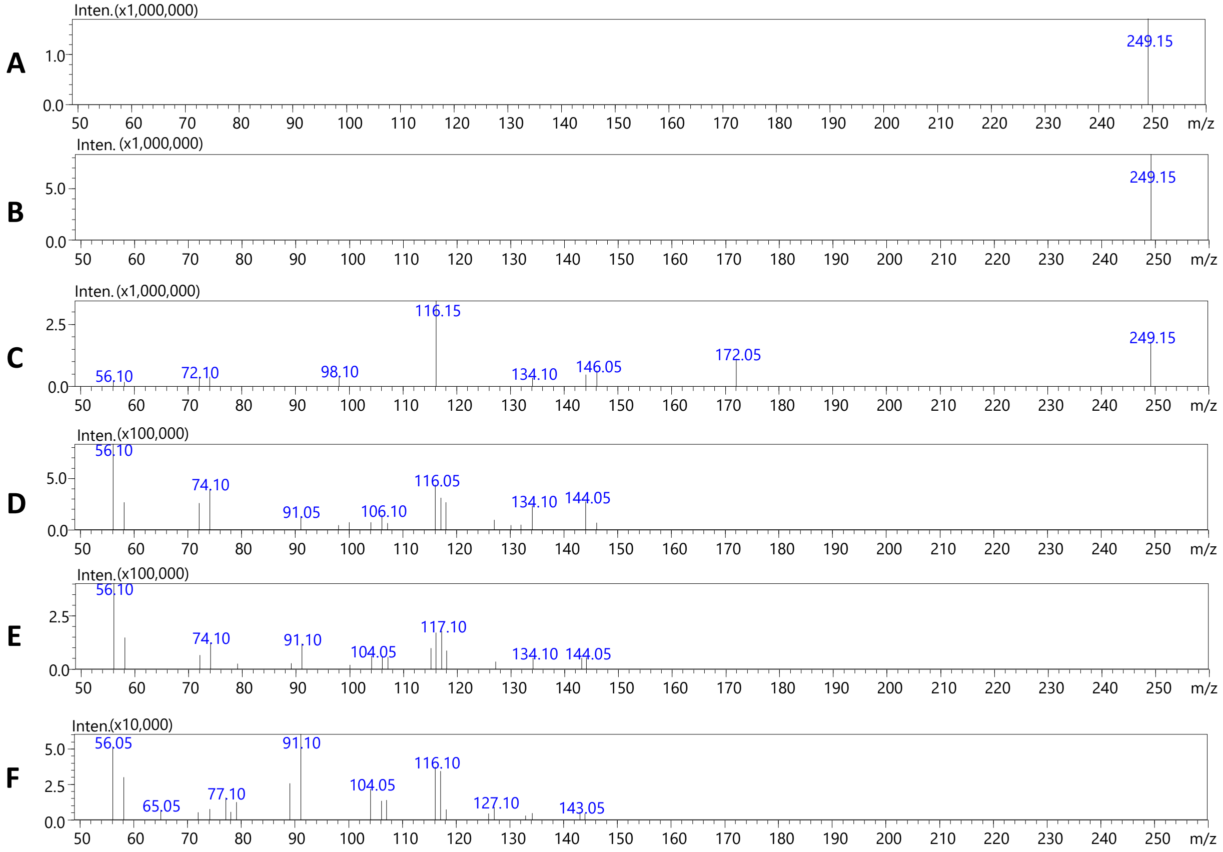

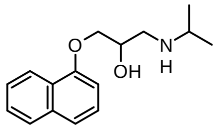

Propranolol

Molecular Formula: $C_{16}H_{21}NO_2$

Formula Weight: 259.349 Da

[M+H]$^+$: 260.164505 m/z

Propranolol

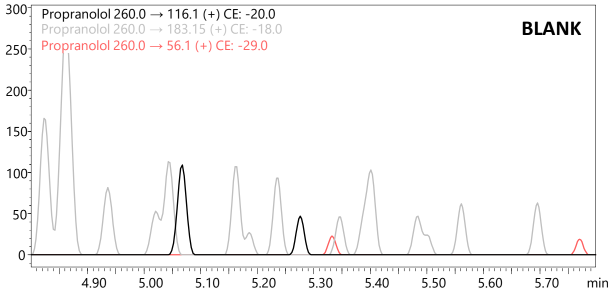

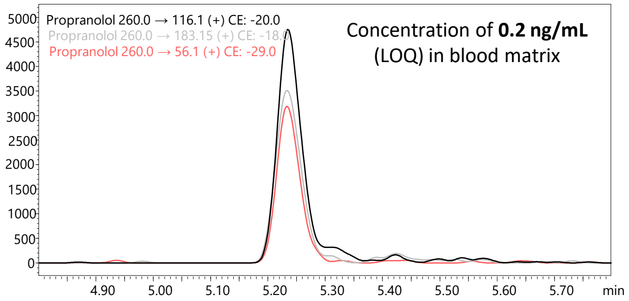

QqQ-MS/MS: Detection of the investigated compounds was achieved with the use of a triple quadrupole mass spectrometer (QqQ, Shimadzu 8050, Kyoto, Japan) in positive mode. The spectrometer was equipped with an electrospray ionization (ESI) source. Fragmentation MS/MS spectra were acquired by conducting a product ion scan experiment at five collision energies CE (**A:** –5, **B:** –10, **C:** –20, **D:** –35, **E:** –40, and **F:** –50 V).

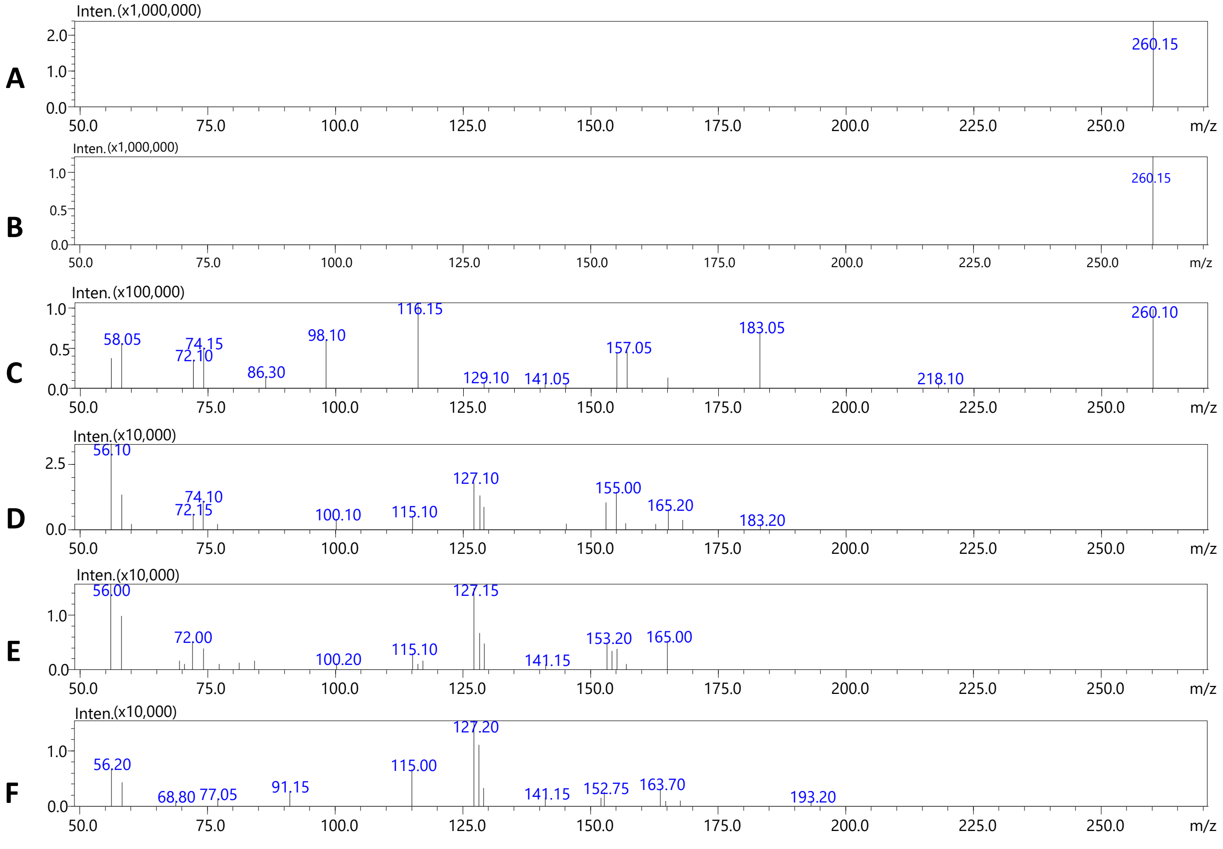

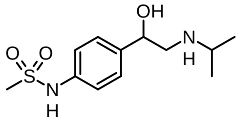

Sotalol

Molecular Formula: $C_{12}H_{20}N_2O_3S$

Formula Weight: 272.363 Da

[M+H]$^+$: 273.126739 m/z

Sotalol

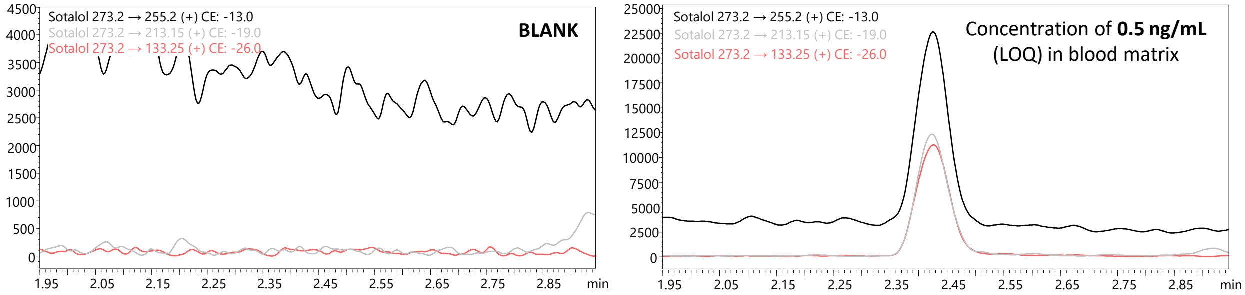

QqQ-MS/MS: Detection of the investigated compounds was achieved with the use of a triple quadrupole mass spectrometer (QqQ, Shimadzu 8050, Kyoto, Japan) in positive mode. The spectrometer was equipped with an electrospray ionization (ESI) source. Fragmentation MS/MS spectra were acquired by conducting a product ion scan experiment at five collision energies CE (**A:** –5, **B:** –10, **C:** –20, **D:** –35, **E:** –40, and **F:** –50 V).

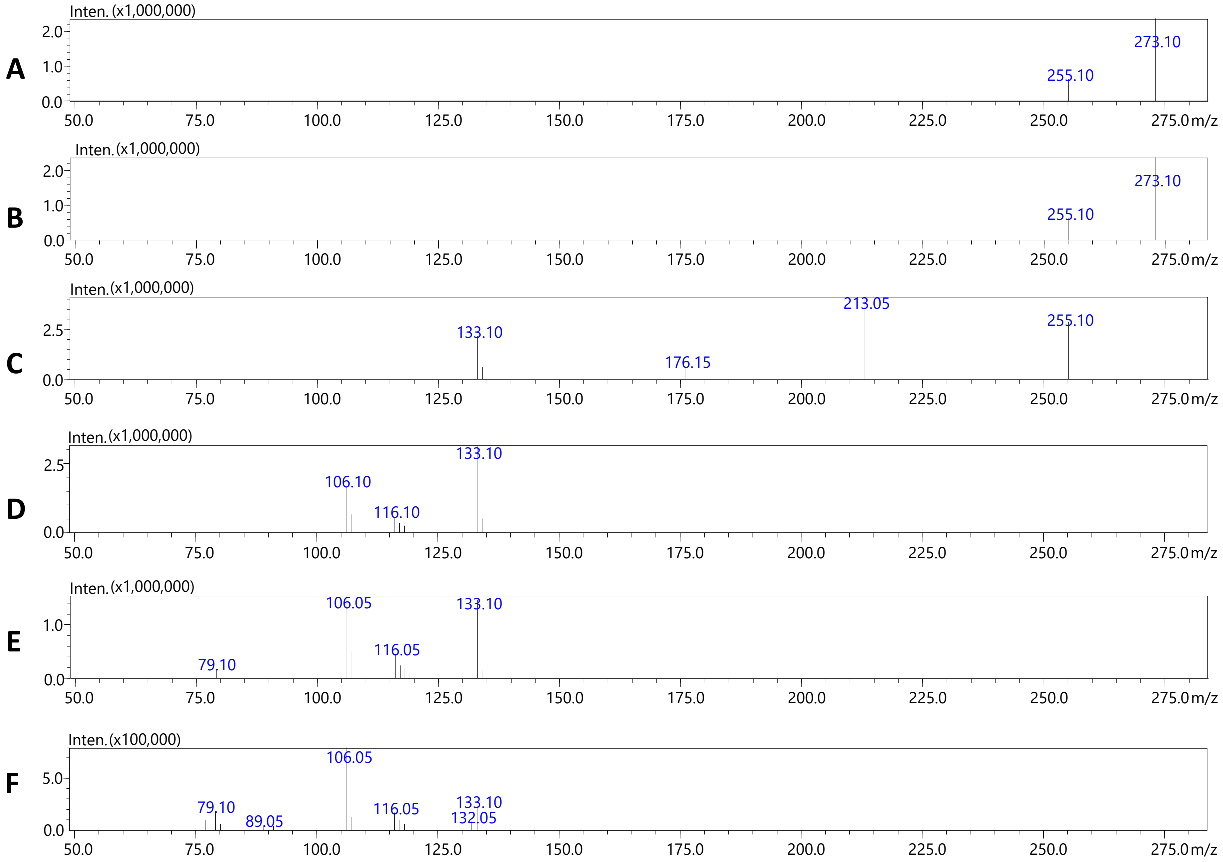

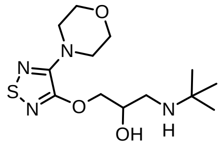

Timolol

Molecular Formula: $C_{13}H_{24}N_4O_3S$

Formula Weight: 316.42 Da

$[M+H]^+$: 317.164187 m/z

Timolol

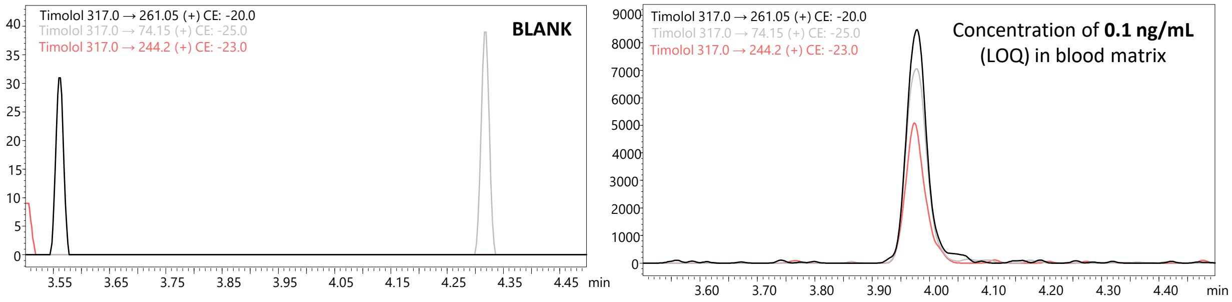

QqQ-MS/MS: Detection of the investigated compounds was achieved with the use of a triple quadrupole mass spectrometer (QqQ, Shimadzu 8050, Kyoto, Japan) in positive mode. The spectrometer was equipped with an electrospray ionization (ESI) source. Fragmentation MS/MS spectra were acquired by conducting a product ion scan experiment at five collision energies CE (**A:** –5, **B:** –10, **C:** –20, **D:** –35, **E:** –40, and **F:** –50 V).

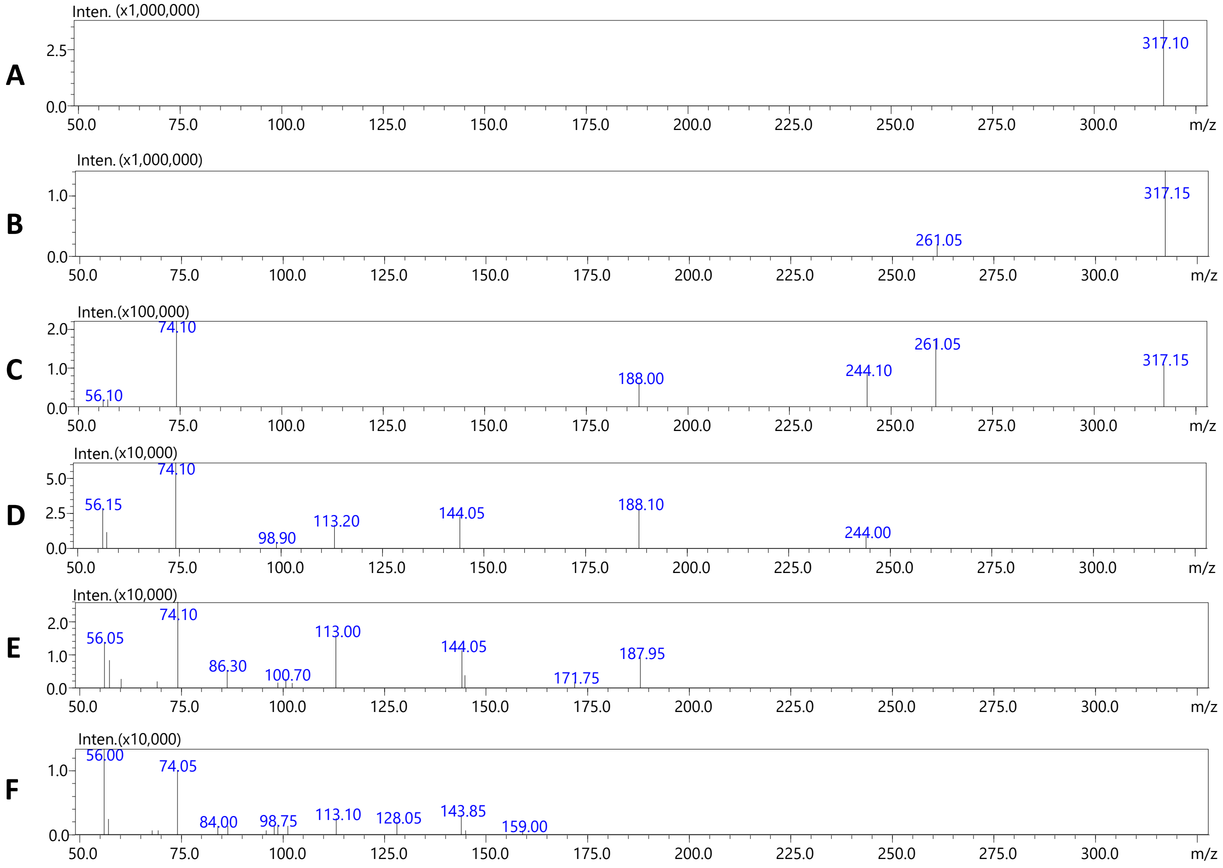

Supplement: Supplementary file 1 [file molecules-29-04585-s001.zip › molecules-3212719-supplementary.pdf]
